# Network Analysis Combining Proteomics and Metabolomics Reveals New Insights Into Early Responses of *Eucalyptus grandis* During Rust Infection

**DOI:** 10.3389/fpls.2020.604849

**Published:** 2021-01-07

**Authors:** Alline Sekiya, Felipe Garbelini Marques, Thiago Falda Leite, Thais Regiani Cataldi, Fabricio Edgar de Moraes, Ana Lúcia Mendes Pinheiro, Mônica Teresa Veneziano Labate, Carlos Alberto Labate

**Affiliations:** Laboratório Max Feffer de Genética de Plantas, Departamento de Genética – Escola Superior de Agricultura Luiz de Queiroz, Universidade de São Paulo, Piracicaba, Brazil

**Keywords:** plant-pathogen interaction, fungus development, microscopy, *Austropuccinia psidii*, LC-MS metabolomics, proteomics, time-course, WGCNA

## Abstract

Eucalyptus rust is caused by the biotrophic fungus, *Austropuccinia psidii*, which affects commercial plantations of Eucalyptus, a major raw material for the pulp and paper industry in Brazil. In this manuscript we aimed to uncover the molecular mechanisms involved in rust resistance and susceptibility in *Eucalyptus grandis*. Epifluorescence microscopy was used to follow the fungus development inside the leaves of two contrasting half-sibling genotypes (rust-resistance and rust-susceptible), and also determine the comparative time-course of changes in metabolites and proteins in plants inoculated with rust. Within 24 h of complete fungal invasion, the analysis of 709 metabolomic features showed the suppression of many metabolites 6 h after inoculation (hai) in the rust-resistant genotype, with responses being induced after 12 hai. In contrast, the rust-susceptible genotype displayed more induced metabolites from 0 to 18 hai time-points, but a strong suppression occurred at 24 hai. Multivariate analyses of genotypes and time points were used to select 16 differential metabolites mostly classified as phenylpropanoid-related compounds. Applying the Weighted Gene Co-Expression Network Analysis (WGCNA), rust-resistant and rust-susceptible genotypes had, respectively, 871 and 852 proteins grouped into 5 and 6 modules, of which 5 and 4 of them were significantly correlated to the selected metabolites. Functional analyses revealed roles for photosynthesis and oxidative-dependent responses leading to temporal activity of metabolites and related enzymes after 12 hai in rust-resistance; while the initial over-accumulation of those molecules and suppression of supporting mechanisms at 12 hai caused a lack of progressive metabolite-enzyme responses after 12 hai in rust-susceptible genotype. This study provides some insights on how *E. grandis* plants are functionally modulated to integrate secondary metabolites and related enzymes from phenylpropanoid pathway and lead to temporal divergences of resistance and susceptibility responses to rust.

## Introduction

*Eucalyptus* ssp. is a genus highly utilized in commercial forestry, with more than 700 species involved in many industrial agribusinesses (EMBRAPA, 2019). Because of its vigor and rapid growth (Moon et al., 2007), *Eucalyptus grandis* and its hybrids are continuously improved to enhance their capacity to provide raw material for the pulp and paper companies in Brazil, the second largest producer of cellulose in the world (EMBRAPA, 2019). Rust, caused by the biotrophic fungus *Austropuccinia psidii* (*A. psidii*), is one of the most harmful diseases affecting Eucalyptus plantations, and consequently, considerably reduces their productivity, particularly in the first 2 years of plant development (Takahashi, 2002). Since *E. grandis* is commonly susceptible to pathogens, geneticists and breeders have used genomic tools applied to conventional breeding to select new rust-resistant genotypes and enhance the development of the next generation trees (Zamprogno et al., 2008; Boava et al., 2010; Silva et al., 2013; Laia et al., 2015; Butler et al., 2016). However, the antagonist bottlenecks have led researchers to question which genes are expressed and translated into functional proteins (Walley et al., 2013; Jafari et al., 2017; Yewdell and Chaudhuri, 2017). Particularly in plant immune regulation, there has been evidence describing that even the induction of transcripts cannot sufficiently predict temporal changes in protein abundance (Xu et al., 2017).

Over the last years, a considerable amount of knowledge became available concerning the molecular mechanisms governing plant pathogenesis. Recently, Shen et al. (2017) coined the term “early responses” that describes the effects of virulent fungi on host plants within an initial interaction that normally occurs until 24 h after inoculation (hai), when the fungal pathogens completely invade the host tissues. According to these authors, many biochemical reactions initiate in the moment at which pathogens and their hosts come in contact, including protein phosphorylation, ion flux, production of reactive oxygen species (ROS) and other signaling events. Using resources within the literature, Rojas et al. (2014) also divided those plant responses into early and late induced defenses. The first consists of cytoskeletal reorganization (Hardham et al., 2007; Higaki et al., 2011), cell wall fortification (Hardham et al., 2007), ROS generation (Torres, 2010), and phytoalexins biosynthesis (Ahuja et al., 2012), and lately, immune defenses induce transcription of pathogenesis-related (PR) proteins (van Loon et al., 2006) and activation of programmed cell death (PCD) allied to the hypersensitive responses (HR), which limit pathogen spread (Coll et al., 2011). Despite the effects of late responses, plants rely heavily on pathogen detection and subsequent signaling cascades to activate genes to enhance defense and immunity (Andersen et al., 2018), which must take place during the early period following initial inoculation.

As one of the key discoveries related to initial plant reactions to pathogens, the identification of phytoalexin production has advanced our knowledge of plant pathology, since these molecules can confer resistance to several diseases. Most of these interactive molecules are known as secondary metabolites and are capable of being elicited by either, biotic or abiotic stresses. Subsequently, they accumulate in host cells to induce protective effects (Daniel and Purkayastha, 1994). Particularly with respect to the defense response to fungal pathogens, phenolic compounds, which are mostly present in leaves, can play functional roles as antioxidants and antimicrobial activities that contribute to plant disease resistance (Shalaby and Horwitz, 2015; Adandonon et al., 2017; Ullah et al., 2017; Maupetit et al., 2018).

Regardless of the role of phytoalexins, other authors have compared the secondary metabolites of Eucalyptus genotypes to identify potential molecules that improve resistance against rust. Dos Santos et al. (2019) studied the cuticular wax of different species of Eucalyptus and discovered an association between rust-susceptibility and hexadecanoic acid levels in *E. grandis* and *E. phaeotricha*. Comparing the oil composition of Eucalyptus leaves in different stages of maturity, Silva et al. (2020) identified a terpenoid called limonene in resistant leaves, which could be associated with rust resistance. Nevertheless, there has been no additional work investigating metabolic activities associated with the molecular control during rust infection.

Envisioning new strategies for enhancing plant disease resistance, one of the principal purposes of plant-pathogen studies is to understand how plants modulate genes, transcripts, proteins and metabolites to physiologically adapt and respond to pathogen invasion. Hence, researchers have used network analyses to group co-expressed genes, integrate omics datasets and reveal new insights into plant physiology (Walley et al., 2013; Ployet et al., 2019), as well as plant response to stress (El-Sharkawy et al., 2015; Yuan et al., 2018; Budzinski et al., 2019; Song et al., 2019).

Aiming to briefly understand the molecular mechanisms surrounding the early interaction between *E. grandis* and *A. psidii*, we describe a detailed time-course microscopy study to characterize the specific events that occur during the development of the *A. psidii* infection in both, resistant and susceptible genotypes. Once defined, time intervals were used to determine molecular details of the early resistance and susceptible responses of *E. grandis* to rust infection. Microscopic analysis confirmed that *A. psidii* completely invades susceptible host tissues within 24 hai, while no pathogen progression was detected in resistant plants. Molecular analyses revealed temporal differences in the metabolomic profiles between the two contrasting genotypes and associated those changes with a proteomic network. Comparative analyses showed that rust resistance depends on temporal control of metabolites and related enzymes from phenylpropanoid pathway, which is mediated by proteomic changes in photosynthesis, oxidative homeostasis and response to stress, predominantly induced from 12 hai. Conversely, due to the lack of continuous or progressive metabolite response after 12 hai, pathogen attack caused rust susceptibility by weakening of the plant immune system. Even though these changes could also consider secondary effects of the experiments, as all omics data, the analyses enabled us to have some clues of rust-responsive molecules and introduce a new biology-based approach for the early *E. grandis* × *A. psidii* interaction.

## Materials and Methods

### Experimental Materials and *A. psidii* Inoculation

One hundred and eight plantlets, kindly provided by Suzano Papel e Celulose, originated from a segregating population of half siblings, from seeds of the BRASUZ tree used by the Joint Genome Institute (JGI)^1^ to sequence the complete genome of *E. grandis*, were used to select the contrasting genotypes, rust-resistant and rust-susceptible. The plantlets were inoculated with a suspension of *A. psidii* spores (10^5^ mL^–1^) containing 0.2% Tween 20 solution of the isolate of the strain MF1. To ensure favorable conditions for fungus development, plants were packed into plastic bags to maintain high humidity inside and kept in a controlled growth chamber (Conviron E15) at 20°C in the dark for 24 h. The photoperiod was then changed to 12 h light (200 μmols m^–2^ s^–1^) and 12 h dark at 24 hai, and the plastic bags were opened at 48 hai. MF1 was previously isolated from a mono pustule formed in a leaf of a highly susceptible, non-commercial, clone of *E. grandis* (M09D1), used for spore propagation. MF1 was obtained from a population of spores collected in commercial plantations in the State of São Paulo, Brazil. Following fungus inoculation, plantlets were evaluated daily for symptoms and classified according to the level of infection as proposed by Junghans et al. (2003). Five plants having high levels of infection (class S3, according to Junghans et al., 2003) and six plants showing no signal of infection (S0) were selected, cloned and reinoculated again in a following experiment. The selection this time proceeded with a higher pressure of spores (10^6^ spores mL^–1^ in 0.2% Tween 20 solution). From this experiment, we were able to select five susceptible and five resistant genotypes, which were then cloned for further experimentation. The selected genotypes were then checked using molecular markers to select two contrasting genotypes as closest genetically as possible. The selected genotypes were R3, which is completely resistant to *A. psidii* MF1 infection, while the S4 genotype is sensitive to the pathogen.

Twelve plants from each genotype were then prepared for a new experiment following the conditions described above. Six plants were inoculated with MF1 spores at a density of 10^5^ mL^–1^ in 0.2% Tween 20 solution and other six control plants were mock-inoculated with 0.2% of Tween 20 solution. Eleven days after inoculation (dai), no disease symptoms were detected in control and inoculated R3 plants. Inoculated S4 plants, however, displayed pustule formation on their leaves 11 dai. DNA-specific amplification of *A. psidii* (Bini et al., 2018) was possible at 3 dai for both inoculated genotypes (Supplementary Figure S1).

To detail the steps of fungus development in both genotypes, whole inoculated leaves were collected at 0, 3, 6, 12, 24, 72 hai and 6, 9, and 12 dai. For molecular analyses of plant responses to rust infection, young leaves were sampled at 0, 6, 12, 18, and 24 hai. Total metabolites were extracted from leaves of each plant separately, while total proteins were obtained from leaves of two plants pooled in a sample. This resulted in six and three biological replicates for metabolomics and proteomics, respectively. For the purpose of combining these omics datasets, analyses were performed using the mean of the metabolite replicates corresponding to each protein sample and the relative abundance of both datasets (ratios of inoculated/mock-inoculated plants).

### Determination of Developmental Stages of *A. psidii* via Epifluorescence Microscopy

All steps were prepared using filter papers with different solutions to avoid spore detachment from leaves during submersion, according to Leite et al. (2013). First, leaves were fixed/bleached with acetic acid:ethanol (1:3) solution for 24 h, rehydrated with water for 4 h and kept in lacto-glycerol solution (lactic acid, glycerol and water 1:1:1 v:v:v). Leaves were then transferred to another filter paper containing boiled KOH solution (1 M) for 10 min and stained with 0.1% calcofluor. Images were taken with an epifluorescence microscope (Zeiss Axioslop 2) using the blue excitation filter (BP 450–490 nm), a beamsplitter (FT 510 nm) and a green barrier filter (Lo 515 nm).

### LC-MS Metabolomics

Approximately 25 mg of leaf powder was ground using a vibration mill (Retch MM400) with tungsten carbide beads for 1 min at 20 Hz. Samples were homogenized with a 500 μL chloroform: water: methanol (1:1:6 v:v:v) solution containing 50 pmol of quercetin (Sigma-Aldrich) (internal standard). The mixture was sonicated (UltraCleaner 1600A, Unique) for 15 min at 4°C, centrifuged (Centrifuge 5415R—Eppendorf) at 16,000 g and 6°C for 10 min, and the supernatant was filtered (Millex/PVDF, 0.22 μm of porosity) to remove contaminants.

Metabolite samples were analyzed in a Q-TOF Ultima-API mass spectrometry, using an electrospray ionization (ESI) source, coupled to an Acquity UPLC HSS T3 (Waters, Corp., Milford, United States). Aliquots of 5 μL of samples were injected onto a reverse-phase column (1.0 × 150 mm, 1.8 μm, Acquity Waters) and two eluents were used as mobile phase: A (100% water containing 0.1% formic acid) and eluent B (100% acetonitrile containing 0.1% formic acid). The gradient used was: 95% A and 5% B for 6 min, 25% A and 75% B for 6 min, 5% A and 95% B for 1 min, as described by Schaker et al. (2017). Voltage was set at 3 kV and 35 kV for capillar and cone, respectively. Temperatures of the ESI-source and desolvation were set at 150°C and 450°C, respectively, and nitrogen flow rates were 50 L h^–1^ in the cone and set 550 L h^–1^ at the source. Using MassLynx 4.1 software (Waters), data were acquired in negative and positive ion mode in a mass range from 100 to 1,000 m/z.

The processing and interpretation of the data obtained by Mass Spectrometry were performed in MassLynx 4.1 (Waters) for alignments of chromatograms, noise exclusion, deconvolution and detection of the intensity of metabolites.

### Statistical Analysis and Metabolite Selection

Statistical analyses of time-series datasets were performed with MetaboAnalyst 4.0 Software (Chong et al., 2018). All data were log transformed and pareto scaled for the following analyses.

In order to evaluate effects of spore inoculation on plants over the time, for each genotype, we performed a 2-way ANOVA using “group” (mock-inoculated and inoculated) and “time” (time-points) as two factors. Significant metabolites were clustered using Euclidean distance and Ward method to plot heatmaps. To investigate effects of “group vs. time interaction,” the differential abundances of metabolites were calculated using the contrast of mock-inoculated vs. inoculated plants for each genotype within each time-point by *t*-test. Results were considered statistically significant when FDR-adjusted *p* < 0.05.

For genotype comparison over the time-course, we used relative abundances of metabolites (rates of inoculated/mock-inoculated values) to perform a PCA for “genotype” (resistant-R3 and susceptible-S4) and “time” (time-points) factors, and analyze the data to detect metabolites that can be used to differentiate rust-resistant and rust-susceptible genotypes throughout the period considered. Metabolites were selected using ANOVA Simultaneous Component Analysis (ASCA) (Smilde et al., 2005) for “genotype vs. time interaction” prominent effects (leverage > 0.9 and Squared Predicted Error < 0.05) and Multivariate Empirical Bayes Analysis (MEBA) (Tai and Speed, 2006) for the first 50 ranking metabolites. The ASCA model was validated with the permutation test (100×), as described by Vis et al. (2007).

LC-ESI-MS/MS analyses of the selected metabolites were performed in the same ionization conditions previously described, and the fragmentation was carried out using collision energies ranging between 15 and 40 eV. We used the Human Metabolite Database (HMDB)^2^ for features identification, considering the aducts [M-H-] (mass error < 0.05 Da) and the *in silico* fragmentation was done with the software ACD/MS structure ID suíte (ACD/Labs, Toronto—Canada) and manually checked. The values of “Spectrum Assigned,” which refers to a percentage of matches in fragmentation similarities, were used to define the best hits of chemical classes attributed to the selected metabolites.

### Proteomics Shotgun Label-Free

Leaf samples (100 mg) were ground using a vibration mill (Retch MM400) with tungsten carbide beads for 1 min at 20 Hz, and were homogenized in 0.8 mL of protein extraction buffer [0.5M Tris-HCl pH 7.5; 0.7M Sucrose; 0.1M Potassium Chloride; 50 mM EDTA; 1mM PMSF; 2% (v/v) β-mercaptoethanol e 1% (m/v) PVPP]. After, 0.8 mL of saturated phenolic solution in Tris-HCl pH 7.5 was added, samples were centrifuged at 10,000 g and 4°C for 30 min. The supernatants were collected and used to repeat this procedure three more times. Proteins were precipitated in 1.2 mL of 0.1 M ammonium acetate in methanol and the pellet was washed with the same solution (two times) and acetone (one time). After the last centrifugation step at 10,000 g and 4°C for 30 min, pellets were dried and proteins were resuspended in 0.4 mL solubilization buffer (7M Urea, 2M Thiourea, 10 mM DTT and 0.4% v/v Triton X-100). Proteins in the supernatant were desalted in 50 mM ammonium bicarbonate buffer (pH 8.5) using an Amicon 3 kDa filter (Millipore), and were quantified using the Bradford method (Bradford, 1976). The quality of protein samples was evaluated using a 12% polyacrylamide gel stained with Comassie Blue G250, and bovine serum albumin was used as an internal standard.

For each sample, 50 μg of proteins were added to 25 μL 2% (v/v) RapiGest SF (Waters) and incubated at 80°C for 15 min. Then, samples were reduced in 2.5 μL 100 mM dithiothreitol (DTT) for 30 min at 60°C and alkylated in 2.5 μL 100 mM iodoacetamide (IAA) for 30 min in the dark. Proteins were digested in 10 μL 50 ng/μL trypsin at 37°C for 16 h, and the reaction was stopped using 10 μL 5% trifluoroacetic acid (TFA). Samples were centrifuged at 14,000 g at 6°C for 30 min, and the peptide-containing supernatant was transferred to another tube to be concentrated using a SpeedVac (Concentrator 5301, Eppendorf). Dried peptides were resuspended in 50 μL 0.1% TFA, purified using reverse-phase micro columns (Reverse phase Zip-Tip C18, Millipore) and dried.

Samples were then resuspended in 32 μL 20 mM pH10 ammonium formate with 8 μL of the 100 fmol μL^–1^ internal standard (P00489. rabbit glycogen-phosphorylase). Peptides were sequenced in a Synapt G2 HDMS mass spectrometer (Waters, Manchester, United Kingdom), connected to UPLC NanoAcquity (2D technology, Waters). In the first dimension, peptides were separated using an XBridge BEH 130 C18 column that was 5 μm (300 μm × 50 mm) (Waters, Manchester, United Kingdom), using a 3–45% gradient of solvent B [0.1% (v/v) ACN], and captured using a C18 symmetry column (5 μm, 180 μm × 20 mm) (Waters, Manchester, United Kingdom). Separation in the second dimension was carried out using an HSS T3 column (1.8 μm, 75 μm × 100 mm) (Waters, Manchester, United Kingdom), and a 7–40% binary gradient of acetonitrile in 0.1% (v/v) and formic acid.

Data acquisition was performed with a Q-TOF Synapt MS, with a nanolockspray font in a positive mode (Waters, Manchester, United Kingdom). The MS run was calibrated with 200 fmol μL^–1^ of Glu1 ([M + 2H]2 + = 785,84206 Daltons), which was also used for lock mass. Mass spectra were processed with the ProteinLynx GlobalServer (PLGS) Program, version 3.0.3, using the protein database with 46,280 proteins of *Eucalyptus grandis* available on Phytozome v13 2.0^3^ (accessed on 03/10/2020). Processing parameters included automatic tolerance of precursors and ion-products and required a minimum of three corresponding ion-fragments per peptide, minimum of seven corresponding ion-fragments per protein, minimum of two corresponding peptides per protein, possible cleavage error of trypsin, carbamidometilation of cysteine with fixed modification and methionine oxidation as variable modifying factors (FDR = 1%).

For protein identification and quantification, spectral intensities were calculated using the stoichiometric method, with an internal standard analyzed with MSE and normalized with the PLGA auto-normalization function. The sequence and abundance of peptides were determined based on the mean values of the three most abundant peptides identified from data obtained from the three biological replicates assessed. FDR values were determined using a reverse database search, which was automatically created by the PLGS 3.0.3 program. Only proteins with confidence levels higher than 95% that were identified and quantified at least in two replicates were considered for subsequent analytical steps.

The mass spectrometry data was deposited to the ProteomeXchange Consortium via the PRIDE (Vizcaíno et al., 2016) partner repository with the dataset identifier PXD021280.

### WGCNA Network Analysis

The WGCNA R package was used to build protein networks based on protein profiles of both resistant-R3 and susceptible-S4 genotypes over the period of 24 hai, identifying protein modules (groups of proteins with similarities in abundance patterns along the time) with minimum size of 80 proteins, and analyze the correlation between protein modules and selected metabolites. The relative abundance of proteins was used to construct networks of positive correlations (signed network) for both genotypes at all time-points. Soft thresholds were chosen by powers (7 for resistant-R3 genotype and 6 for susceptible-S4 genotype) that suit a maximum approximation of a scale free topology distribution and small connectivity corresponding to high correlations between protein pairs (Supplementary Figure S2). Since WGCNA was performed for each genotype separately, module names initiated by “R” belong to the resistant-R3 genotype and “S” belong to the susceptible-S4 genotype. The network of protein modules was visualized in Cytoscape, in which each node represented a protein and the length of each edge represented the strength of the correlation between a protein pair. These plots were used to observe the proximity between proteins in modules and the network topology obtained for each genotype. To identify groups of proteins that could potentially explain the occurrence of selected metabolites, eigenvalues of protein modules were calculated as the first principal component to represent each protein group and were correlated to the metabolites. Correlations were considered significant when *p* < 0.05. All WGCNA analyses were performed in R, according to Langfelder and Horvath (2008).

### Functional Analysis

To better enhance our understanding of mechanisms influencing resistance and susceptibility of genotypes when challenged by the biotrophic fungi, all protein modules were functionally analyzed. The gene ontology (GO) of all proteins in modules was determined using AgriGO v2.0^4^ and the *E. grandis* database. GO-terms were considered enriched when FDR < 0.05.

For modules that were significantly correlated to the metabolites, the sequences of proteins were used in KAAS-KEGG^5^ to map metabolic pathways containing metabolite-related enzymes.

## Results

Evidence of rust disease was observed in inoculated S4 plants appearing as yellow pustules containing spores on leaf surfaces (both adaxial and abaxial sides) at 11 dai, while inoculated R3 leaves displayed mild flecking (chlorosis spots) response for resistance mediated by HR. Controls (mock-inoculated) did not show disease symptoms (Supplementary Figures S1A,B). The presence or absence of the pathogen in inoculated and mock-inoculated plants was also confirmed by PCR using pathogen-specific primers of *A. psidii* at 3 dai (Supplementary Figures S1C,D).

### Epifluorescence Microscopic Analysis of *A. psidii* Development

**FIGURE 1:**
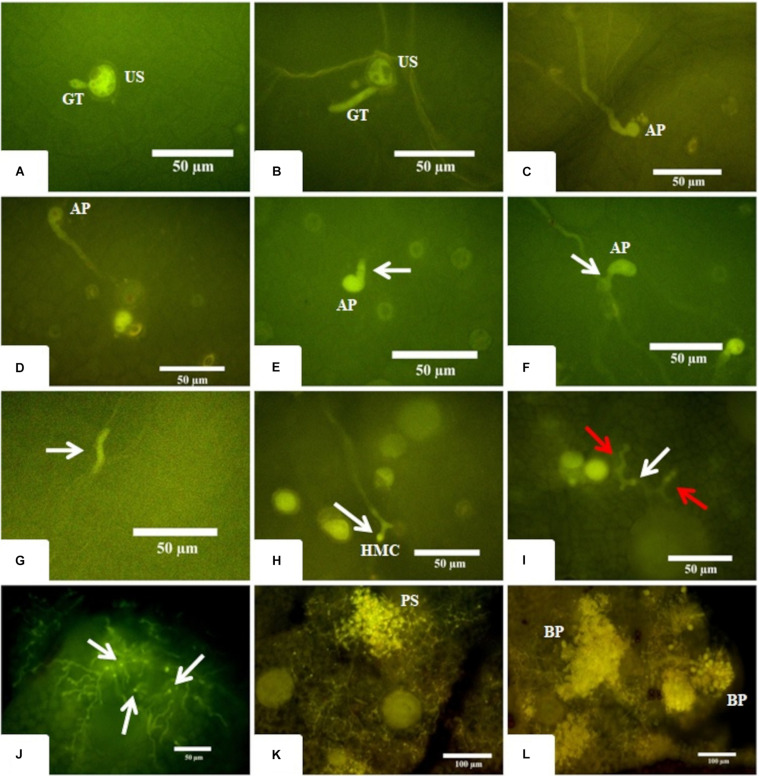
Microscopy images of fungus development in both resistant-R3 and susceptible-S4 genotypes. **(A,B)** The beginning of the germination process where a single unbranched germ tube (GT) emerges from the urediniospore (US) at 3 hai in the resistant-R3 and susceptible-S4 genotypes, respectively. **(C,D)** The apressorium (AP) formation at 6 hai in the resistant-R3 and susceptible-S4 genotype, respectively. **(E,F)** The penetration process at 12 hai in resistant-R3 and susceptible-S4 genotypes, respectively. White arrows show the subepidermal vesicule arising from the AP only in susceptible-S4 genotype. **(G)** At 24 hai, just some hyphae fragments (white arrow) can be found in the resistant-R3 genotype. **(H)** The Haustorium Mother Cell (HMC) can be seen at 24 hai in the susceptible-S4 genotype. **(I)** The beginning of the colonization phase at 72 hai in susceptible-S4 genotype. Red arrows show the secondary hyphae. White arrow shows the HMC. **(J)** At 6 dai in the susceptible-S4 genotype, the fungus has colonized large areas of the leaf and numerous HMCs (white arrows) can be seen. **(K)** At 9 dai, the mesophyll are completely colonized and small pustules (PS) are being formed in the susceptible-S4 genotype. **(L)** After 12 dai, the vast majority of the pustules had burst (BP) through the leaf epidermis spilling new urediniospores (US) onto the leaf surface of the susceptible-S4 genotype.

To better understand the temporal development of *A. psidii* in R3 and S4 plants, inoculated leaves were collected at different time-points post-treatment and examined using epifluorescence microscopy (Figure 1). Until 12 hai, the development of *A. psidii* occurred similarly on both genotypes. Rust urediniospores germinated at 3 hai, formed appressoria at 6 hai and penetrated inside the leaf tissue at 12 hai, but subepidermal vesicle was only visible in the S4 genotype (Figures 1A–F). Following 24 hai, haustorium mother cells were observed in S4 leaves, while only hyphal fragments were noticed and thereafter no evidence of the pathogen progression was detected in R3 (Figures 1G,H).

Overall, little or no fungal growth was observed in S4 plants between 24 and 72 hai, indicating that the fungus was apparently in a latent state. However, at 72 hai it was possible to observe the secondary hyphae colonizing the mesophyll (Figure 1I). With 6 dai several parts of the leaf’s mesophyll were intensely colonized (Figure 1J) and, at 9 dai it was completely colonized with the hyphae converging to form the first pustules (Figure 1K). Finally, at 12 dai a plethora of large pustules burst is observed, covering the leaves with urediniospores (Figure 1L).

This result showed that *A. psidii* was able to cross the first physical barrier of the plant to penetrate the leaves of both R3 and S4 genotypes. However, the progression through all stages of *A. psidii* development was reported exclusively in S4 leaves within 24 hai. On the other hand, responses of R3 plants highly diverged from S4 leaves at 12 hai, since the subepidermal vesicle of the fungus was not detected. At 24 hai the R3 defense system was probably induced, and the pathogen had no more progression.

From the eight time-points analyzed using epifluorescence microscopy, five were chosen to well characterize the stages within the infection process to compare the molecular responses of R3 and S4 after rust inoculation: 0 h established as the control, 6 hai where the spores have already germinated and appressoria were produced, 12 hai where the pathogen penetrated the leaf tissue and forms subepidermal vesicles, 18 hai where R3 potentially activates the defense response (between 12 and 24 hai) and 24 hai the defense process was installed in R3 and the fungus formed haustorium in S4.

### Analysis of Metabolomic Profiles for Metabolites Selection

LC-MS metabolomic analyses were performed in positive and negative ion mode. However, in positive mode we were able to detect few metabolite features, whereas in negative mode we detected 709 features, from all treatments. Therefore, we decided to consider only the results obtained in negative mode.

**FIGURE 2:**
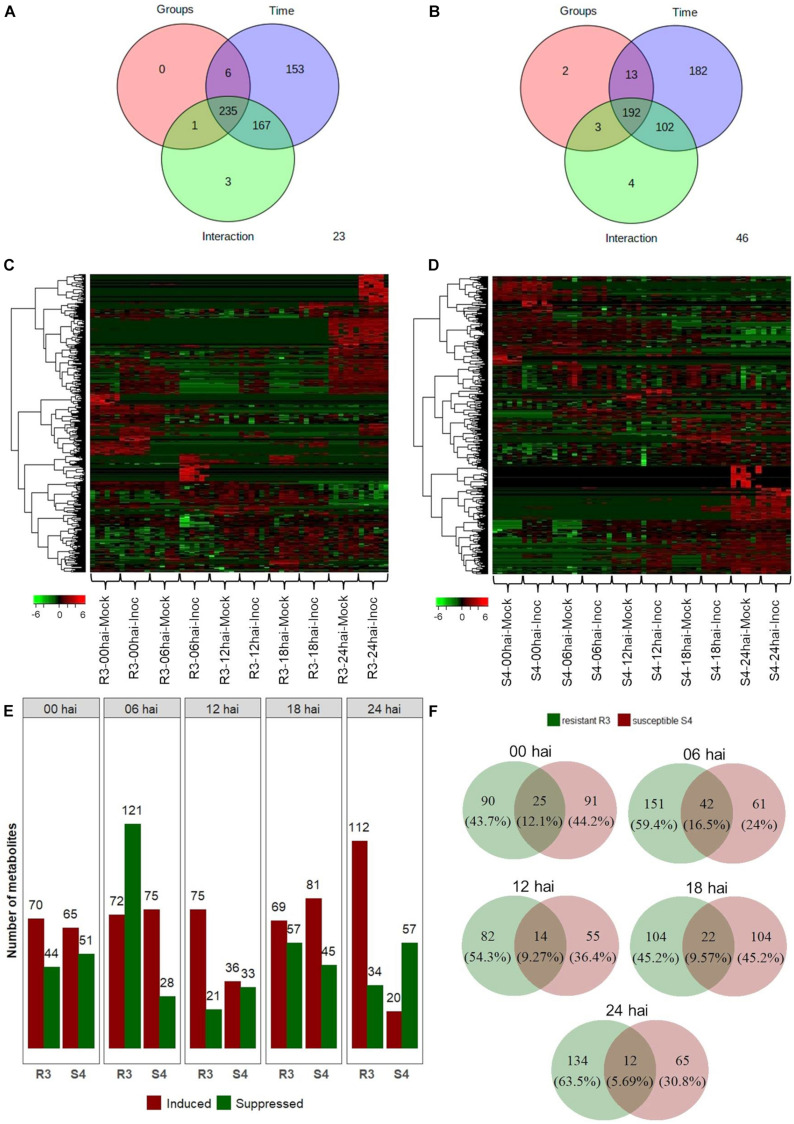
Two-way ANOVA for 709 metabolite features obtained from “groups” (inoculated and mock-inoculated plants) and “time” (00, 06, 12, 18, and 24 hai time-points) treatments of the resistant-R3 and susceptible-S4 genotypes of *E. grandis* in response to rust infection. Venn diagrams showing the number of significant features for “groups,” “time,” and “groups vs. time interaction” effects: **(A)** resistant-R3 and **(B)** susceptible-S4 genotypes. Heatmaps of significant features: **(C)** resistant-R3 and **(D)** susceptible-S4 genotypes. **(E)** The number of differential metabolite features of the resistant R3 and suceptible-S4 genotypes by contrasting inoculated and mock-inoculated groups within each time-point by *t*-test. Bars in green indicate down-regulated metabolites and bars in red represent up-regulated metabolites. **(F)** Venn diagrams of differential metabolite features in contrasting genotypes at each time-point considered. Green circles represent the resistant-R3 genotype and red circles represent the susceptible-S4 genotype. Results of statistical analyses were considered significant when FDR-adjusted *p* < 0.05.

The 2-way ANOVA showed that, for the resistant-R3 and susceptible-S4 genotypes, most of the metabolite features were significantly affected by both “group,” “time,” and “group vs. time interaction” effects (Figures 2A,B). These results demonstrated that the artificial inoculation of *A. psidii* uredospores induced abundance changes in metabolite features of both genotypes along the time. However, many other metabolite features were exclusively significant for “time” effects, which highlighted that some variations were caused by other temporal effects, such as the circadian rhythm, exposure to light absence and/or other environmental condition. All these metabolite features were observed in heatmaps illustrating hierarchical clustering based on their abundance values (Figures 2C,D). Apparently, in the resistant-R3 genotype, the metabolite profile of mock-inoculated and inoculated plants had some differences at all time-points, but changes were more remarkable at 6 and 24 hai. On the other hand, in the susceptible-S4 genotype, abundance patterns of some metabolite features became closer between mock-inoculated and inoculated plants throughout the time.

To depict effects of “group vs. time interaction,” differential abundance analysis between mock-inoculated and inoculated plants within each time-point revealed distinct metabolite profiles for both genotypes over the time. R3 inoculated plants had increased abundances of 70, 72, 75, 69, and 112 metabolite features and reduced abundances of 44, 121, 21, 57, and 34 metabolite features compared to the R3 mock-inoculated plants at 0, 6, 12, 18, and 24 hai, respectively. In S4 inoculated plants, however, 65, 75, 36, 81, and 20 metabolite features increased in abundance and 51, 28, 33, 45, and 57 decreased in abundances compared to the S4 mock-inoculated plants at 0, 6, 12, 18, and 24 hai, respectively (Figure 2E). Thus, we observed that at 0 hai, the rates of positive and negative changes in abundance of metabolite features (1.79 for resistant-R3 and 1.32 for susceptible-S4) were similar in both genotypes. However, production of many metabolite features was abruptly suppressed in R3 plants at 6 hai, which was followed by a greater number of metabolites that increased in abundance at subsequent time-points, mostly evident at 24 hai. On the other hand, the metabolic profiles of S4 plants had more metabolites increasing than decreasing in abundance at 6, 12, and 18 hai, but there were more features being suppressed at 24 hai. Analysis using Venn diagrams also showed that, at 0 and 6 hai, more than 12% of the differentially abundant features identified from R3 and S4 overlapped (Figure 2F). This value decreased to approximately 9% at 12 and 18 hai, and 5.69% at 24 hai. These results indicated that at 6 hai, part of the metabolites of R3 and S4 genotypes were inversely regulated activating divergent responses observed after 12 hai, with differences that were most striking at 24 hai.

**FIGURE 3:**
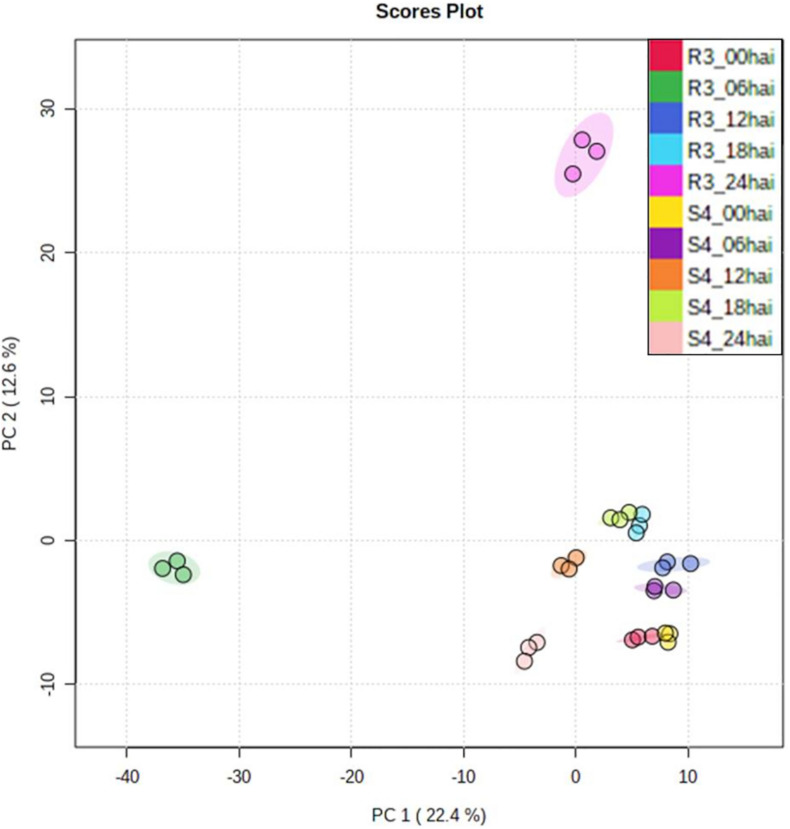
PCA of genotype- and time-point-based groups. Values were represented using relative abundances (rates of inoculated/mock-inoculated values) of genotypes and time-points.

Using relative abundances of metabolite features (rates of inoculated/mock-inoculated values) to compare resistant-R3 and susceptible-S4 responses against *A. psidii* infection, the PCA revealed that the metabolite profiles at different time-points in S4 plants were more similar than R3 genotype (Figure 3). For R3 plants, the metabolite profiles of each time-point were scattered and a major distinction at 6 and 24 hai was observed. To identify metabolites whose abundances are most representative of temporal differences between R3 and S4 genotypes, ASCA analysis was performed, allowing the detection of 27 prominently affecting metabolite features for “genotype vs. time interaction” (Supplementary Figure S3), which were compared to the list of the 50 highest ranking features from the MEBA analysis (Supplementary Table S1). MS/MS analyses showed that these features had correspondence with different metabolites (Supplementary Material 1). The statistical and fragmentation analyses resulted in 16 selected metabolites, mostly predicted as secondary metabolites derived from the phenylpropanoid pathway (Table 1). The identification of this biosynthetic pathway was consistent with this pathosystem, since *A. psidii* is a biotrophic fungus which can induce the biosynthesis of phenylpropanoids in plants as a metabolic response (Doehlemann et al., 2008; Hossain et al., 2018; Correr et al., 2020). Five metabolites were considered as “unknown” because of the low MS intensity required for fragmentation or no correspondence in MS/MS metabolites of HMDB.

**TABLE 1:** Biochemical classes of the 16 selected metabolites based on MS/MS analysis using searches of the m/z values in Human Metabolite Database (HMDB), considering [M-H]-ion mode and molecular weight tolerance < 0.05 Da.

***m/z***	**HMDB_code**	**Spectrum assigned**	**Class**	**Super class**
169.0847	HMDB0030471	61.7	Heteroaromatic compounds	Organoheterocyclic compounds
191.052				
207.0997	HMDB0029872	82.2	Phenol ethers	Benzenoids
	HMDB0034991	83.7	Benzene and substituted derivatives	Benzenoids
	HMDB0040225	84.1	Benzene and substituted derivatives	Benzenoids
301.0362	HMDB0015039	91.9	Benzene and substituted derivatives	Benzenoids
399.1279	HMDB0061023	48.7	Benzothiazepines	Organoheterocyclic compounds
	HMDB0033258	45.7	Furanoid lignans	Lignans, neolignans and related compounds
	HMDB0040556	44.9	Flavonoids	Phenylpropanoids and polyketides
487.147	HMDB0035860	78.0	Steroids and steroid derivatives	Lipids and lipid-like molecules
489.0887	HMDB0128306	38.1	Depsides and depsidones	Phenylpropanoids and polyketides
505.0924	HMDB0033894	57.7	Flavonoids	Phenylpropanoids and polyketides
	HMDB0037352	58.4	Flavonoids	Phenylpropanoids and polyketides
533.1794	HMDB0031584	36.4	Diarylheptanoids	Phenylpropanoids and polyketides
533.1832	HMDB0133430	58.5	Diarylheptanoids	Phenylpropanoids and polyketides
615.0893	HMDB0037367	66.8	Coumarans	Organoheterocyclic compounds
	HMDB0033593	68.5	Flavonoids	Phenylpropanoids and polyketides
784.0677				
865.1768	HMDB0036339	41.5	Benzopyrans	Organoheterocyclic compounds
915.0872				
935.0737				
985.1147				

**FIGURE 4:**
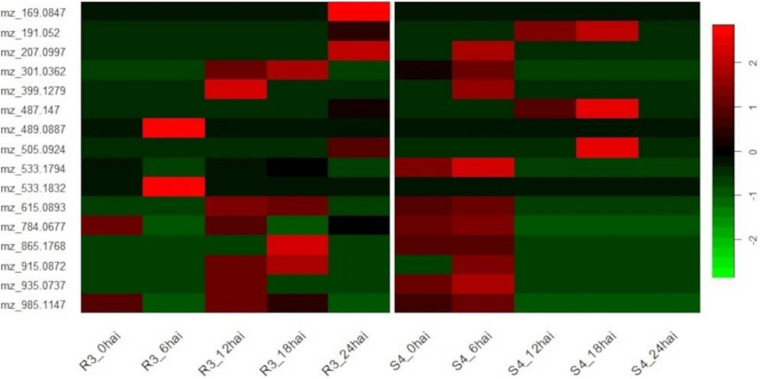
Heatmap of genotype-specific (resistant-R3 and susceptible-S4) time-course for 16 metabolites selected by ASCA (ANOVA simultaneous component analysis) producing prominent effects for “genotype vs. time-point” interactions and MEBA (multivariate empirical Bayes analysis) to rank features, and evaluated by MS/MS analysis.

Comparing the time-course of selected metabolites synthesized in different genotypes, we noted a degree of temporal divergence with respect to the time-points at which the metabolites were produced. Most of the metabolites were induced before 12 hai in S4 plants and from 12 hai in R3 plants. None were detected in S4 plants at 24 hai (Figure 4).

### WGCNA Protein Modules and Their Correlation to the Selected Metabolites

Shotgun label-free proteomic analysis identified and quantified a total of 1007 proteins from all treatments. As recommended by WGCNA developers (Langfelder and Horvath, 2008), proteins were not filtered by differential abundance to be clustered in modules, according to their corresponding gene co-expression values. Based on relative abundance, 871 and 852 proteins from respective R3-resistant and S4-susceptible genotypes were grouped into 5 and 6 modules defined by “R” and “S” color-names, with size ranging from 102 to 226 proteins, and represented by their eigenvalue, as the principal component of each protein group (Supplementary Figure S4). However, 94 and 48 proteins did not fit to any pattern of respective R3 and S4 module profiles and were not used for the following analyses.

**FIGURE 5:**
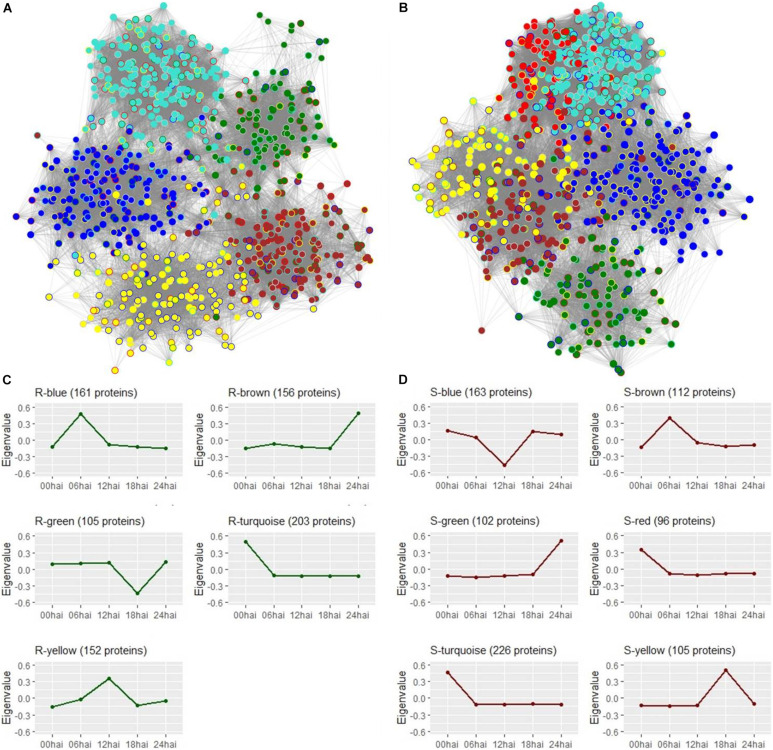
Protein networks visualization from Cytoscape software. **(A)** Resistant-R3 genotype with 777 protein nodes from 5 modules represented by colors and 34,469 connecting edges. Module colors of the resistant-R3 genotype were used to fill nodes and the border were colored with the module color of the same proteins in the susceptible-S4 genotype. **(B)** Susceptible-S4 genotype with 804 protein nodes from 6 modules represented by colors and 49,803 connecting edges. Module colors of the susceptible-S4 genotype were used to fill nodes and the border were colored with the module color of the same proteins in the resistant-R3 genotype. In **(A,B)**, connection strength is proportional to edge length and border nodes with gray color have no protein correspondence with the alternative genotype. **(C)** Temporal profiles of resistant-R3 protein modules using eigenvalues. **(D)** Temporal profiles of susceptible-S4 protein modules using eigenvalues.

Protein networks created in WGCNA were visualized in the Cytoscape^®^ environment to observe the connections between proteins within each module. Proteins were represented by nodes and the strength of connections between protein pairs were indicated by the length of each edge. To investigate differences in network topologies and rearrangement of the proteins between the two contrasting genotypes, module colors of the present genotype were used to fill nodes and the border were colored by the module color of the alternative genotype. For both genotypes, we were able to note that proteins from the same module were relatively near to each other (Figures 5A,B). Nonetheless, we also identified strong connections between proteins from S-red and S-turquoise neighboring modules of the susceptible-S4 genotype, confirming high proximities among these two different protein modules. Comparing genotypes, we could note that proteins were differentially distributed across “R” and “S” modules resulting in distinct characterization of the resistant-R3 and susceptible-S4 network topologies. Looking at the border colors of nodes, we observed that only the R-turquoise and S-turquoise modules, defined by the same color, overlapped many proteins; and also that, R-yellow module had more proteins that belong to the S-blue module than the others of the susceptible-S4 genotype, and vice-versa. The other “R” and “S” protein modules were composed by proteins from different color modules of the alternative genotype, which confirmed their divergence in protein distribution through the networks.

Protein time-courses of each module were apparently responsible for specific time-points, but in some cases, a slight variation was also observed at others (Figures 5C,D). R3 plants had changes in protein levels of the R-turquoise module at 0 hai; R-blue module at 6 hai; R-yellow at 12 hai; R-green module at 18 hai; R-brown modules at 24 hai. On the other hand, S4 plants had changing proteins at 0 hai in S-turquoise and S-red modules; at 6 hai in S-brown module; at 12 hai in S-blue module; at 18 hai in S-yellow module; and at 24 hai in S-green module. The high similarity of S-turquoise and S-red time-courses probably explains their strong connection observed in the S4 protein network. Only R-green and S-blue modules reported low abundances of proteins compared to the other time-points.

**FIGURE 6:**
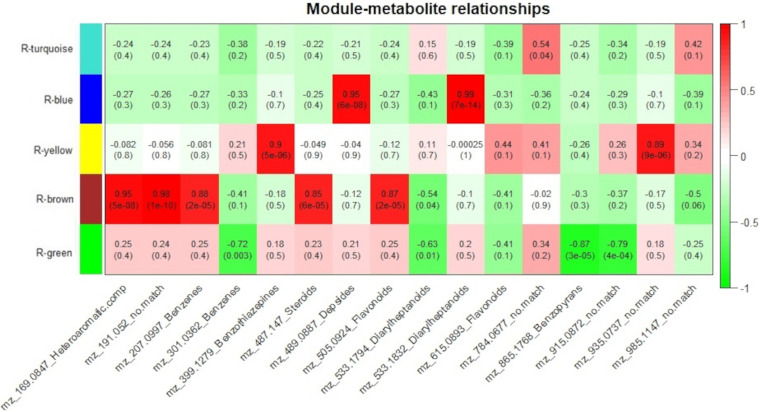
Resistant-R3 correlation analysis of protein modules and selected metabolites. Protein modules are represented by colors at the left side and selected metabolites are represented by their m/z and chemical classes at the bottom. Correlation values and *p*-values are present inside the rectangles. Red and green colors indicate positive and negative correlations, respectively. Correlations were considered significant when *p* < 0.05.

**FIGURE 7:**
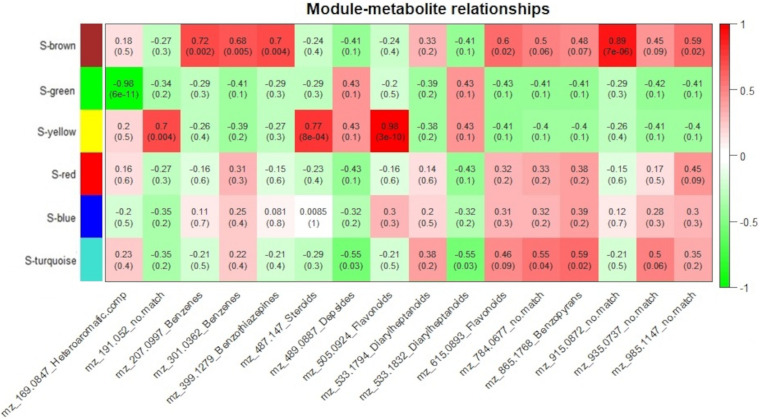
Susceptible-S4 correlation analysis of protein modules and selected metabolites. Protein modules are represented by colors at the left side and selected metabolites are represented by their m/z and chemical classes at the bottom. Correlation values and *p*-values are present inside the rectangles. Red and green colors indicate positive and negative correlations, respectively. Correlations were considered significant when *p* < 0.05.

To measure the relationship between protein groups and metabolites, for each genotype, module eigenvalue values were correlated to selected metabolites, which resulted in 15 significant correlations (*p* < 0.05) between 5 protein modules and 14 metabolites for the resistant-R3 genotype and 14 significant correlations (*p* < 0.05) between 4 protein modules and 14 metabolites for the susceptible-S4 (Figures 6, 7). Two metabolites had no significant correlation to protein modules in the resistant-R3 and susceptible-S4 genotypes. Most of the significant metabolite-protein relationships were positively correlated. Only the R-Brown and R-green module of the resistant-R3, the S-green and S-turquoise modules of the susceptible-S4 had negatively correlated metabolites.

For the resistant-R3 genotype, R-turquoise protein module was positively correlated to m/z 784.0677 (*no match*) metabolite; R-blue protein module was positively correlated to m/z 489.0887 (Depsides) and m/z 533.1832 (Diarylheptanoids) metabolites; R-yellow protein module was positively correlated to m/z 399.1279 (Benzothiazepines) and m/z 935.0937 (*no match*) metabolites; R-brown protein modules was positively correlated to m/z 169.0847 (Heteroaromatic compounds), m/z 191.052 (*no match*), m/z 207.0997 (Benzenes), m/z 487.147 (Steroids), m/z 505.0924 (Flavonoids) metabolites and negatively correlated to m/z 533.1794 (Diarylheptanoids) metabolite; and R-green protein module was negatively correlated to m/z 301.0362 (Benzenes), m/z 533.1794 (Diarylheptanoids), m/z 865.1768 (Benzopyrans) and m/z 915.0872 (*no match*) metabolites (Figure 6). Only m/z 615.0893 (Flavonoids) and m/z 985.1147 (*no match*) were not explained by any R-module.

The Susceptible-S4 genotype had S-brown protein module positively correlated to m/z 207.0997 (Benzenes), m/z 301.0362 (Benzenes), m/z 399.1279 (Benzothiazepines), m/z 615.0893 (Flavonoids), m/z 915.0872 (*no match*), and m/z 985.1147 (*no match*) metabolites; S-green protein modules was negatively correlated to m/z 169.0847 (Heteroaromatic compounds); S-yellow protein modules was positively correlated to m/z 191.052 (*no match*), m/z 487.147 (Steroids) and m/z 505.0924 (Flavonoids) metabolites; and S-turquoise protein modules was positively correlated to 784.0677 (*no match*) and m/z 865.1768 (Benzopyrans) metabolites and negatively correlated to m/z 489.0887 (Depsides) and m/z 533.1832 (Diarylheptanoids) metabolites (Figure 7). The metabolites m/z 533.1794 (Diarylheptanoids) and m/z 935.0937 (*no match*) had no corresponding S-module, as well as S-red and S-blue modules were not correlated to any metabolite.

As we expected, most of the selected metabolites were correlated to protein modules with higher abundance of proteins after 12 hai in resistant-R3 genotype (R-brown and R-green modules) and before 12 hai in susceptible-S4 genotype (S-brown and S-turquoise modules) (Figures 6, 7).

### Functional Biology of Protein Modules and Their Metabolite-Related Pathways

To elucidate a biological purpose of the protein changes involved in the temporal resistance or susceptibility of *E. grandis* to rust disease, the GO terms of all protein modules were analyzed in AgriGO and main results were summarized in Table 2.

**TABLE 2:** Summary of important GO-terms* associated with protein modules of resistant-R3 and susceptible-S4 genotypes.

	**Resistant-R3**	**Susceptible-S4**
0 hai	**R-turquoise module**	**S-turquoise and S-red modules**
	– Metabolic process (GO:0008152)	– Metabolic process (GO:0008152)
	– Generation of precursor metabolites and energy (GO:0006091)	– Generation of precursor metabolites and energy (GO:0006091)
	– Catabolic process (GO:0009056)	– Catabolic process (GO:0009056)
	– Nucleotide metabolic process (GO:0009117)	– Nucleotide metabolic process (GO:0009117)
	– Purine nucleotide metabolic process (GO:0006163)	– Purine nucleotide metabolic process (GO:0006163)
	– Translation (GO:0006412)	– Translation (GO:0006412)
	– Photosynthesis (GO:0015979)	– Photosynthesis (GO:0015979)
	– Cellular homeostasis (GO:0019725)	– Cellular homeostasis (GO:0019725)
6 hai	**R-blue module**	**S-brown module**
	– Metabolic process (GO:0008152)	– Generation of precursor metabolites and energy (GO:0006091)
	– Generation of precursor metabolites and energy (GO:0006091)	– Catabolic process (GO:0009056)
	– Catabolic process (GO:0009056)	– Nucleotide metabolic process (GO:0009117)
	– Nucleotide metabolic process (GO:0009117)	– Purine nucleotide metabolic process (GO:0006163)
	– Purine nucleotide metabolic process (GO:0006163)	– Translation (GO:0006412)
	– Translation (GO:0006412)	
	– Cellular homeostasis (GO:0019725)	
12 hai	**R-yellow module**	**S-blue module**
	– Metabolic process (GO:0008152)	– Metabolic process (GO:0008152)
	– Generation of precursor metabolites and energy (GO:0006091)	– Generation of precursor metabolites and energy (GO:0006091)
	– Catabolic process (GO:0009056)	– Catabolic process (GO:0009056)
	– Nucleotide metabolic process (GO:0009117)	– Nucleotide metabolic process (GO:0009117)
	– Purine nucleotide metabolic process (GO:0006163)	– Purine nucleotide metabolic process (GO:0006163)
	– Photosynthesis (GO:0015979)	– Photosynthesis (GO:0015979)
	– Response to oxidative stress (GO:0006979)	
	– Oxidation-reduction process (GO:0055114)	
18 hai	**R-green module**	**S-yellow module**
	– Metabolic process (GO:0008152)	– Metabolic process (GO:0008152)
	– Generation of precursor metabolites and energy (GO:0006091)	– Catabolic process (GO:0009056)
	– Catabolic process (GO:0009056)	– Response to oxidative stress (GO:0006979)
	– Nucleotide metabolic process (GO:0009117)	– Oxidation-reduction process (GO:0055114)
	– Purine nucleotide metabolic process (GO:0006163)	– Translation (GO:0006412)
	– Translation (GO:0006412)	
24 hai	**R-brown module**	**S-green module**
	– Metabolic process (GO:0008152)	– Metabolic process (GO:0008152)
	– Generation of precursor metabolites and energy (GO:0006091)	– Generation of precursor metabolites and energy (GO:0006091)
	– Catabolic process (GO:0009056)	– Catabolic process (GO:0009056)
	– Nucleotide metabolic process (GO:0009117)	– Nucleotide metabolic process (GO:0009117)
	– Purine nucleotide metabolic process (GO:0006163)	– Purine nucleotide metabolic process (GO:0006163)
	– Photosynthesis (GO:0015979)	
	– Translation (GO:0006412)	
	– Response to stress (GO:0006950, GO:0006979)	
	– Oxidation-reduction process (GO:0055114)	

*GO-terms were considered enriched when FDR-adjusted p < 0.05. The whole list of all enriched GO-terms associated with these protein modules are available in Supplementary Tables S2–S12.

In early response of plants to rust, both genotypes had enriched GO-terms related to changes in primary metabolism (i.e., GO:0008152, metabolic process; GO:0044281, small molecule metabolic process; GO:0006091, generation of precursor metabolites and energy, GO:0009056, catabolic process) and processing of genetic information (i.e., GO:0009117, nucleotide metabolic process; GO:0006163, purine nucleotide metabolic process; GO:0006753, nucleoside phosphate metabolic process) in almost all “R” and “S” modules of the resistant-R3 and susceptible-S4 genotypes respectively (Supplementary Tables S2–S12). At the beginning of infection, R-turquoise and S-turquoise modules, mostly related to high abundance of proteins 0 hai, were similarly enriched for other GO-terms, such as *translation* (GO:0006412), *photosynthesis* (GO:0015979) and *cellular homeostasis* (GO:0019725) (Supplementary Tables S2, S8). Although R-blue and S-brown modules did not shared many proteins at 6 hai, both kept enriched GO-terms associated with *translation* (GO:0006412), but only R-blue module was enriched for *cellular homeostasis* (GO:0019725) yet (Supplementary Tables S3, S9).

At 12 hai, when R-yellow and S-blue modules inversely regulated some common proteins, many GO-terms related to photosynthesis regulation (i.e., GO:0015979, photosynthesis; GO:0034357, photosynthetic membrane; GO:0009579, thylakoid) were enriched for both protein modules (Supplementary Tables S4, S10). This may indicate that the high abundance of photosynthesis-related proteins at this time-point had a functional role in inducing resistance to rust disease, as well as the low abundance of the same proteins could lead to rust-susceptibility. Moreover, R-yellow module also had GO-terms for *response to oxidative stress* (GO:0006979) and *oxidation-reduction process* (GO:0055114), which were only enriched for proteins with high abundance at 18 hai of the S-yellow module (Supplementary Table S11). At this time-point, R-green module had down-regulated proteins for translation-related GO-terms (i.e., GO:0006412, translation; GO:0043043, peptide biosynthetic process; GO:0006518, peptide metabolic process), also found in S-yellow module (Supplementary Tables S5, S11).

At 24 hai, resistant-R3 and susceptible-S4 genotypes exhibited major functional differences between each other. Highly abundant proteins of R-brown module were enriched for many GO-terms linked to photosynthesis (i.e., GO:0015979, photosynthesis; GO:0019684, photosynthesis, light reaction; GO:0009765, photosynthesis, light harvesting; GO:0009579, thylakoid; GO:0009521, photosystem), translation (GO:0006412, translation; GO:0043043, peptide biosynthetic process), response to stress (GO:0006950, response to stress; GO:0006979, response to oxidative stress) and antioxidant processes (GO:0055114, oxidation-reduction process; GO:0016491, oxidoreductase activity; GO:0016491, antioxidant activity) (Supplementary Table S6). Otherwise, proteins of the S-green module had no relevant GO-term when compared to the other S-modules (Supplementary Table S12).

During the time-course many proteins in R3 plants related to plant defense were produced. Before 12 hai, induced proteins that were related to stress (i.e., Stress responsive alpha-beta barrel domain protein; heat shock protein 70; Pathogenesis-related thaumatin superfamily protein) and oxidative balancing [i.e., *ascorbate peroxidase 4; – NAD(P)-linked oxidoreductase superfamily protein; lipoxygenase 2; thioredoxin family protein; Peroxidase superfamily protein*] were found in R-blue module (Supplementary Table S3). Subsequently, proteins for correlated metabolites (i.e., *dehydroquinate dehydratase, putative/shikimate dehydrogenase, putative; cinnamyl-alcohol dehydrogenase; Chalcone-flavanone isomerase family protein; Chalcone and stilbene synthase family protein*) and many others strongly associated to plant oxidative responses (i.e., *ascorbate peroxidase 1; Thioredoxin superfamily protein; catalase 2; copper/zinc superoxide dismutase 1; Eucgr.I01408—ascorbate peroxidase 3; ascorbate peroxidase 1; NAD(P)-linked oxidoreductase superfamily protein; thioredoxin-dependent peroxidase 1; glutathione peroxidase 6; Glutaredoxin family protein*) were present in the R-yellow and R-brown modules (Supplementary Tables S4, S6).

On the other hand, susceptible-S4 plants also had proteins associated with the selected metabolites (i.e., *Chalcone-flavanone isomerase family protein; dehydroquinate dehydratase, putative/shikimate dehydrogenase, putative*) and oxidative balancing [*NAD(P)-linked oxidoreductase superfamily protein; ascorbate peroxidase 1; thioredoxin-dependent peroxidase 1; Thioredoxin superfamily protein; NAD(P)-linked oxidoreductase superfamily protein; Glutaredoxin family protein; copper/zinc superoxide dismutase 2*] mainly in S-turquoise, S-red, and S-brown modules strongly associated to the time-point before 12 hai (Supplementary Tables S7–S9), but it was less intensive than the resistant-R3 responses. Further, proteins related to stress (*Stress responsive alpha-beta barrel domain protein; heat shock protein 70; heat shock protein 91; heat shock protein 70B*) were lately observed in S-yellow and S-green modules (Supplementary Tables S11, S12).

**FIGURE 8:**
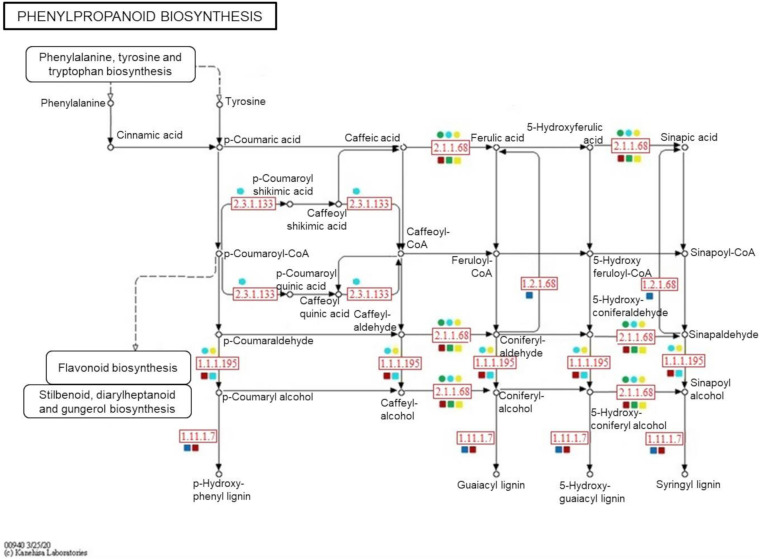
Simplified representation of KEGG phenylpropanoid pathway. Enzymes identified in protein modules are highlighted in red according to their response within each genotype and protein-module assessed. Resistant-R3 and susceptible-S4 genotypes are indicated with square and circle shapes, respectively. Protein modules were represented by their module colors. e.c. numbers correspond to: e.c. 2.1.1.68—*caffeate O-methyltransferase enzyme*; e.c. 1.1.1.195—*cinnamyl-alcohol dehydrogenase*; e.c. 1.2.1.68—*coniferyl-aldehyde dehydrogenase*; e.c. 1.11.1.7—*peroxidase*; e.c. 2.3.1.133—*shikimate O-hydroxycinnamoyltransferase.* Enzymes and related precursors and products, which are not present in “R” and “S” protein modules, were removed.

**FIGURE 9:**
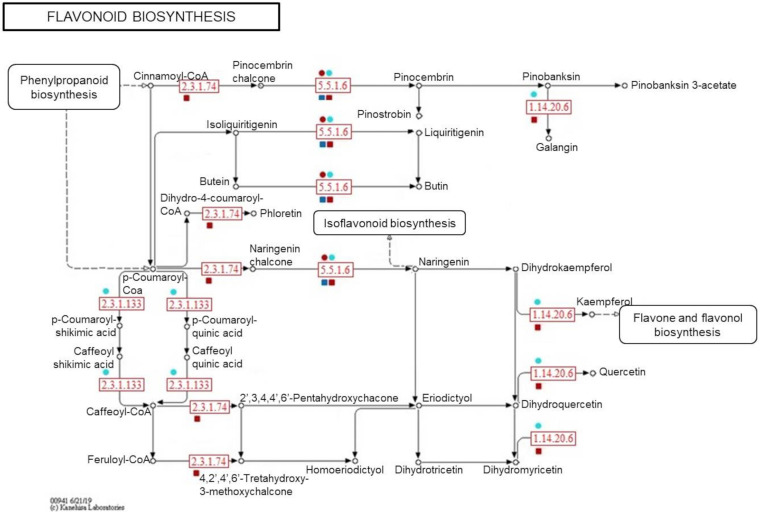
Simplified representation of KEGG flavonoid pathway. Enzymes identified in protein modules are highlighted in red according to their response within each genotype and protein-module assessed. Resistant-R3 and susceptible-S4 genotypes are indicated with square and circle shapes, respectively. Protein modules were represented by their module colors. e.c. numbers correspond to: e.c. 5.5.1.6—*chalcone-flavanone isomerase*; e.c. 1.14.20.6—*flavonol synthase*; e.c. 2.3.1.74—*chalcone synthase*; e.c. 2.3.1.133—*shikimate O-hydroxycinnamoyltransferase.* Enzymes and related precursors and products, which are not present in “R” and “S” protein modules, were removed.

To confirm the metabolite-protein correlations described, 5 and 4 protein-enzymes from significantly metabolite-correlated modules of the genotypes were mapped in respective phenylpropanoid and flavonoid pathways (KEGG) (Figures 8, 9). Both genotypes controlled their levels of the *caffeate O-methyltransferase enzyme* (e.c. 2.1.1.68) and *cinnamyl-alcohol dehydrogenase* (e.c. 1.1.1.195) from phenylpropanoid pathway, and *chalcone-flavanone isomerase* (e.c. 5.5.1.6) and *flavonol synthase* (e.c. 1.14.20.6) from flavonoid pathway along the time. However, only R-modules had *coniferyl-aldehyde dehydrogenase* (e.c. 1.2.1.68) and *peroxidase* (e.c. 1.11.1.7) from phenylpanoid pathway and *chalcone synthase* (e.c. 2.3.1.74) from flavonoid pathway. In the same way, *shikimate O-hydroxycinnamoyltransferase* (e.c. 2.3.1.133) was unique to the S-modules. In general, resistant-R3 genotype accumulated more metabolite-related enzymes in the R-brown module, closely associated with 24 hai; while in susceptible-S4, more enzymes were detected in the S-turquoise module with high abundances of proteins at 0 hai. These findings corroborated with the results we got from metabolomic analysis, in which metabolites involved in the phenylpropanoid pathway showed high abundances before 12 hai in the susceptible-S4 genotype, and after 12 hai in the resistant-R3.

## Discussion

The early responses of plants to pathogen infection are comprised of a wide range of molecular events which may initiate rapidly after plants and pathogens come into contact. Studies have shown that the initial stages of plant-pathogen interactions usually occur within 24 h after inoculation. In this period, pathogens can completely invade host tissues and the efficiency of plant defenses determines whether plants will be susceptible or resistant to infection (Shen et al., 2017).

In general, the stages of rust infection can be categorized into temporal phases corresponding to fungal development in which plants recognize specialized structures and activate defense responses (Hu and Rijkenberg, 1998). Therefore, light microscopy analysis can be a useful method to elucidate the timeframe in which the pathogen growth ceases in resistant plants (Hu and Rijkenberg, 1998; Xavier et al., 2001; Ayliffe et al., 2011; Zhang et al., 2011) and progress in susceptible tissues. We noticed that MF1 urediniospores germinated at 3 hai and facilitated appressorium formation at 6 hai in both R3 and S4 genotypes. Xavier et al. (2001) also observed no differences on *A. psidii* germination and appressorium formation between *E. grandis* rust-divergent genotypes, however, these structures delayed three more hours to be detected in comparison to our study. It is possible that the use of different plant genotypes and fungal isolates explains the variation in timing.

To support pathogen invasion and colonization, chemical compounds produced in fungal appresorium can degrade plant cell wall to be transferred into the subepidermal vesicle, located just inside the host tissue. Then, the penetrating hypha elongates in the leaf parenchyma to invade the host cells and induce haustorium formation, a bulging structure used to absorb nutrients from plants. MF1 fungal isolate was able to penetrate in the leaf mesophyll of both R3 and S4 genotypes at 12 hai, but the subepidermal vesicle was only visible in susceptible plants. Its absence in the R3 genotype revealed the developmental stage in which fungal growth disruption probably occurs. In a similar investigation of *Puccinia recondita* sp. tritici in non-host species, Hu and Rijkenberg (1998) were also unable to detect fungal development after substomatal vesicle formation in sorghum. In addition, only fragments of hyphal structures were observed, and the pathogen had no progression in R3 genotype at 24 hai, at the same time-point *A. psidii* produced haustorium in S4 plants. Xavier et al. (2001) also noticed similar observations of *A. psidii* development in rust-resistant and rust-susceptible genotypes of *E. grandis* within 24 hai. As determined by Shen et al. (2017), for early plant responses to fungal infections, *A*. *psidii* had completely invaded leaf tissues of S4-susceptible plants within 24 hai. During this period, *E. grandis* resistance was established between 12 and 24 hai, initiating just after pathogen penetration in R3 plants.

Since the early molecular events can mostly define either incompatible or compatible interactions of plants and fungal pathogens, the use of multi-omics strategies offer an ideal approach to understand mechanisms underlying plant disease resistance and susceptibility, and consequently, has the potential for the discovery of new molecules or genes to be used to enhance molecular breeding and/or gene-editing programs (Yuan et al., 2018). Despite omics experiments yield hypothetical results concerning the up- and down-regulation of molecules, since they can reflect other secondary effects, researchers have been applied these technologies to uncover the molecular mechanisms involved in plant-pathogen interactions (Cueto-Ginzo et al., 2016; Chong et al., 2018; Pang et al., 2018; Chen et al., 2019; Jia et al., 2020). Based on the time-course established by the study of fungus development, we also report our efforts to compare an integrative metabolomic and proteomic responses of two half-sibling genotypes of *E. grandis* during the early stages of rust infection.

Since plant-pathogen interactions are a spatially dynamic process, the bioactive effects of secondary metabolites on plant immunity depend on their accumulation at the proper concentration, at a specific time and place (Piasecka et al., 2015). During *A. psidii* infection, *E. grandis* genotypes had more than 60 metabolites per genotype and time with differential abundance, but distinctions in metabolite profile were observed between genotypes, even more at 24 hai, when pathogen produced haustorium in susceptible-S4 leaves and had no progression in resistant-R3. The genotype-specific divergence observed regarding the number of induced and suppressed metabolites support the idea that *A. psidii* could weaken the defense system of S4 plants within this period. Santos et al. (2020) also suggested that the down-regulation of genes associated with plant defenses at 24 hai is mediated by *A. psidii* effectors in rust-susceptible *E. grandis* plants. This may lead to the suppression of pathogen-induced changes in secondary metabolism. Although the authors did not find many differentially expressed genes in the rust-resistant genotype at 24 hai, the progressive induction of effective metabolites in R3 plants was able to disrupt fungi development just after its penetration.

Our analysis revealed 16 important metabolites that were significantly different in both genotypes in specific time-points, to explain temporal differences between resistant and susceptible plants inoculated with *A. psidii*. The results identified putative targets for breeding efforts. Most metabolites were chemically classified as flavonoids, benzenoids, diarylheptanoids, coumarans, lignans, and other phenolic compounds or derivatives. As a special defense against biotrophic pathogens, phenylpropanoid metabolic pathways are activated in response to shikimate pathway and provide a large array of molecules with antioxidant and/or antimicrobial activities.

As it was reported before, most of the selected metabolites were identified as derivatives of phenylpropanoid pathway, of which three can be potential flavonoids. R3 plants accumulated an increased number of selected metabolites after 12 hai, at the same interval that pathogen penetrated their leaves and then had no more growth. On the other hand, the initial over-accumulation followed by a decreased abundance of selected metabolites just after pathogen penetration in S4 plants suggested that it was an unsuccessful plant strategy used to control its infection process. Alternatively, it could have been a result of the manipulation of plant metabolic pathways by the pathogen to weaken plant defenses and facilitate its development. It is widely known that flavonoids and other phenolic compounds play a key role in plant immunity, but their antimicrobial efficacy depends on both chemical structure and the strain of microorganism (Iranshahi et al., 2015). Scientific reviews have also described the antifungal potential of flavonoids (Treutter, 2006; Iranshahi et al., 2015; Piasecka et al., 2015; Chen et al., 2019), but the understanding of how these biochemical compounds interact with pathogenic microorganisms requires further investigation.

Although there is insufficient information regarding the interaction between metabolites and the pathogen, we aimed to enhance our understanding of how *E. grandis* genotypes molecularly respond to *A. psidii* infection. Using WGCNA, resistant-R3 and susceptible-S4 genotypes had, respectively, 5 and 6 protein modules with particular abundance profiles throughout the time. Therefore, once each protein module was determined to be more responsive to a specific time-point, we paid special attention to temporal-specific responses.

Regarding to the protein network and time-course information, we could consider that the manner of how these proteins were rearranged through the networks had strong relation with the molecular mechanisms that resistant-R3 and susceptible-S4 genotypes responded to the interaction with the pathogen. R-turquoise and S-turquoise modules had common proteins with high abundance at the 0 hai, showing that both genotypes initiated their protein profiles similarly at the beginning of infection. However, R-yellow and S-blue modules, which also shared many proteins, interestingly displayed respective high and low abundances of the proteins at 12 hai. The inverse regulation of proteins related to photosynthesis may elucidate important details of how the genotypes diverged their defense responses and report the time-point when it occurred. ATP, NADPH and carbohydrates derived from photosynthesis reactions have functional roles in plant defenses, since they may lead to the biosynthesis of many compounds, such as salicylic acid (SA), primary and secondary metabolites (Lu and Yao, 2018). Santos et al. (2020) also revealed that rust-resistant eucalypts constitutively overexpress genes involved in photosynthesis compared to rust-susceptible plants, which reinforce that photosynthesis is a key process to unveil the mechanism of plant responses to *A. psidii* infection.

The other protein modules and corresponding time-points had no evident relationship between resistant-R3 and susceptible-S4 genotypes, since their composition were clearly different. This contrast possibly explain the molecular dynamics governing mechanisms that lead to rust-resistance and rust-susceptibility of *E. grandis* plants within 24 hai.

As a typical plant immune response, the generation of ROS triggers multiple resistance strategies against biotrophic pathogens (Passardi et al., 2005). Incompatible interactions usually involve two temporal oxidative bursts for pathogen detection and signaling that lead to HR and PCD, to create a delimited zone that is used to prevent its growth and spread (Barna et al., 2012). Oxidative environments are toxic and unsuitable even for pathogen survival and can inhibit fungal spore germination (Lamb and Dixon, 1997). In addition to these pathogen-targeted responses, ROS also participates in signal transduction and facilitates lignin polymerization to strengthen the cell wall and prevent microbial penetration (Bradley et al., 1992; Passardi et al., 2005; Calderan-Rodrigues et al., 2019). However, to not suffer by their toxic effects, plants must provide a good balance between ROS production and detoxifying mechanisms to activate defense responses avoiding the destruction of their own plant cells by themselves (Decros et al., 2019). Different types of reduction-oxidation proteins and associated GO-terms were present in protein modules associated with potential antimicrobial and/or antioxidant metabolites in both genotypes, but a temporal difference suggest that R3 plants responded more appropriately to the oxidative stress and produced many more proteins involved in oxidative balancing at each time-point than S4 plants. Moreover, only R3 inoculated plants confirmed resistance responses mediated by HR appeared as fleck spots on leaf surfaces at 11 dai, when pustules containing spores ensured successful rust infection in S4 inoculated leaves. Other works have also reported HR in resistant genotypes at 48 hai using staining methods (Xavier et al., 2001) and fleck reactions as a symptom of HR used to phenotype resistance response of Eucalyptus to rust (Junghans et al., 2003).

To support our findings, many stress-related proteins were induced in R3 plants during the first contact to pathogens before 12 hai. Subsequently, even more proteins related to oxidative balancing and those that were associated with the selected metabolites likely enhanced resistance against *A. psidii* after its penetration. In contrast, the high abundance of metabolites and correlated proteins with potential antioxidant and antimicrobial effects at early time-points, and other proteins related to stress and oxidation-reduction processes after 12 hai, were not enough to defend S4 plants against pathogen attack. Possibly, a decompensated temporal control of initial putative antioxidant metabolites and subsequent detoxifying proteins could strongly reduce oxidative species in S4 plants, mitigating key downstream processes of plant defenses. Other works have previously reported that the up-regulation of ROS scavengers could trigger susceptibility to powdery mildew in barley (El-Zahaby et al., 1995).

As another way to understand the molecular relationship between metabolic and proteomic responses of *E. grandis* during *A. psidii* infection, an integrative assessment revealed the temporal modulation of phenylpropanoid and flavonoid enzymes in both genotypes. When challenged by biotrophic pathogens, plants activate metabolic processes to produce salicylic acid (SA), a shikimate-derived phenolic compound produced in late pathogen signaling (Li et al., 2019). The most common mechanism of SA biosynthesis involves the phenylpropanoid pathway, which also originates a myriad of other phenolic compounds. Studies of some fungal diseases have reported the enrollment of this intricate pathway in plant disease resistance. For example, in maize, it was reported to be related to defense against *Ustilago maydis* (Doehlemann et al., 2008), in soybean the pathway mediated resistance to *Rhizoctonia solani* (Copley et al., 2017) and in sorghum it enhanced the defense response against *Colletotrichum sublineolum* (Tugizimana et al., 2019). In *E. grandis*, comparative transcriptomics of contrasting genotypes reveal constitutive expression of genes for response to SA in rust-resistant plants (Santos et al., 2020). Here, a great part of the 16 selected metabolites belong to the phenylpropanoid pathway, of which some were classified as possible flavonoids. During 24 h of *A. psidii* infection, R3 and S4 plants differently modulated both metabolites and related enzymes. As mentioned previously, those were concentrated before 12 hai in susceptible-S4 and after 12 hai in resistant-R3 genotype. Besides that, R3 plants produced two other enzymes from phenylpropanoid pathway that were not noticed in S4 plants. Coniferyl-aldehyde dehydrogenase (e.c. 1.2.1.68) and peroxidase (e.c. 1.11.1.7) enzymes participate in different stages of lignin biosynthesis (Calderan-Rodrigues et al., 2019; Ferro et al., 2020). As a consequence of oxidative effects, these results may suggest that rust-resistance also relies on strengthening the cell wall, while rust-susceptibility is characterized by a defect in temporal strategy that lacks an efficient response against *A. psidii*-specific infection.

## Conclusion

In spite the fact that Eucalyptus species have other mechanisms that enhance rust resistance, combined metabolome and proteome analyses could help us to elucidate differences in the early responses of two half-sibling genotypes of *E. grandis* to rust. In accordance with our temporal investigation of the plant pathosystem, rust-resistance and rust-susceptibility are defined within 24 hai. Microscopy analysis shows that *A. psidii* equally grows in both genotypes until 12 hai, when it penetrates inside leaf mesophyll. After that, progressive pathogen development only occurs in S4 plants while R3 defenses are probably activated. Molecular signatures of R3 and S4 plants also reveal that secondary metabolites and enzymes from phenylpropanoid pathway have distinct profiles in plant responses before and after 12 hai. During pathogen development outside leaves, rust resistance is conditioned by dynamic processes that are mediated by proteins for stress-related before 12 hai. Changes in photosynthesis deliver the energy and precursors required to produce targeted defenses after 12 hai, when an equilibrated distribution of secondary metabolites and related enzymes is crucial for rust resistance. Its association with proteins reveals a role for the modulation of oxidative effects in the enhancement of immune activation against the biotrophic fungus *A. psidii*. On the other hand, the initial accumulation of potential antioxidant metabolites and detoxifying proteins possibly acting as ROS scavengers before 12 hai, and a molecular suppression of photosynthesis at 12 hai, likely induced rust-susceptibility by disabling dependent responses for the secondary metabolism after pathogen penetration. Thus, comparative analyses used to study the early responses of *E. grandis* genotypes by applying integrative metabolomics and proteomics approaches during rust infection enabled us to determine temporal differences in plant responses that lead to either resistance or susceptibility for this plant-pathosystem model, and were able to identify key pathways involving potential metabolites and proteins with roles in the plant immunity.

## Data Availability Statement

The mass spectrometry data was deposited to the ProteomeXchange Consortium via the PRIDE (Vizcaíno et al., 2016) partner repository with the dataset identifier PXD021280.

## Author Contributions

AS designed the research, wrote the manuscript, and performed statistical and network analyses. FGM and TL carried out the experiments. TL provided epifluorescence microscopy images. FGM produced the omics datasets. TC and FEM processed the data. CL conceived and coordinated the study and revised the manuscript. All authors contributed with discussions and approved the final manuscript.

## Conflict of Interest

The authors declare that the research was conducted in the absence of any commercial or financial relationships that could be construed as a potential conflict of interest.

## Footnotes

1 https://phytozome.jgi.doe.gov

2 https://hmdb.ca/

3 https://phytozome.jgi.doe.gov

4 http://systemsbiology.cau.edu.cn/agriGOv2/

5 https://www.genome.jp/tools/kaas/

## Supplementary Material

Supplementary Figure 1 Images of inoculation and control plants at 11 days after inoculation. **(A)** Control R3 and **(B)** S4 plants lack disease symptoms. **(C)** Inoculated R3 plants do not form pustules, but display symptoms of hypersensitive reactions. **(D)** Inoculated S4 plants have pustules covering the leaf surface. An agarose gel containing amplified DNA fragments produced using *A. psidii*-specific primer that confirm the inoculation of **(E)** R3 and **(F)** S4 genotypes.

Supplementary Figure 2 Choosing soft threshold based on power value (numbers in red) for constructing adjacency matrix network. **(A)** Power 7 of resistant-R3 samples showed scale free topology value > 0.8 and mean connectivity < 50 and **(B)** power 6 of susceptible-S4 samples showed scale free topology value close to 0.6 and mean connectivity < 50.

Supplementary Figure 3 Two-factor ASCA (ANOVA simultaneous component analysis) analysis for genotype, time-point and genotype vs. time-point interaction. **(A)** Distribution of metabolite features in which square predict error (SPE) < 0.05 and leverage > 0.9 for genotype for effects of time-point or genotype vs. time-point interactions. Outlier features are present in the upper quadrants (blue sections) and prominently regulated features are shown in the pink quadrant at the bottom right. **(B)** ASCA model validation. Distribution of sums of squares (SSQ) using 100x permutation of groups (genotype and time-points) is shown. Gray columns indicate SSQ distribution of data permutation and red arrows highlight the SSQ of original dataset format, which confirms the model.

Supplementary Figure 4 WGCNA (weighted gene co-expression network analysis) cluster dendrogram of protein modules represented by color bars at the bottom. **(A)** Resistant-R3 genotype had 5 modules from 871 proteins. **(B)** Susceptible-S4 genotype has 6 modules from 852 proteins.

Supplementary Table 1 Top 50 metabolites (*m/z*) identified by multivariate empirical bayes analysis.

Supplementary Table 2 Resistant_0H_R-turquoise.

Supplementary Table 3 Resistant_6H_R-blue.

Supplementary Table 4 Resistant_12H_R-yellow.

Supplementary Table 5 Resistant_18H_R-green.

Supplementary Table 6 Resistant_24H_R-brown.

Supplementary Table 7 Susceptible_0H_S-red.

Supplementary Table 8 Susceptible_0H_S-Turquoise.

Supplementary Table 9 Susceptible_6H_S-brown.

Supplementary Table 10 Susceptible_12H_S-blue.

Supplementary Table 11 Susceptible_18H_S-yellow.

Supplementary Table 12 Susceptible_24H_S-green.
