# Supplementary figures and images for: Network Analysis Combining Proteomics and Metabolomics Reveals New Insights Into Early Responses of Eucalyptus grandis During Rust Infection
Source: Front Plant Sci. 2021 Jan 7;11:604849. doi: 10.3389/fpls.2020.604849 (PMC7817549; doi:10.3389/fpls.2020.604849)

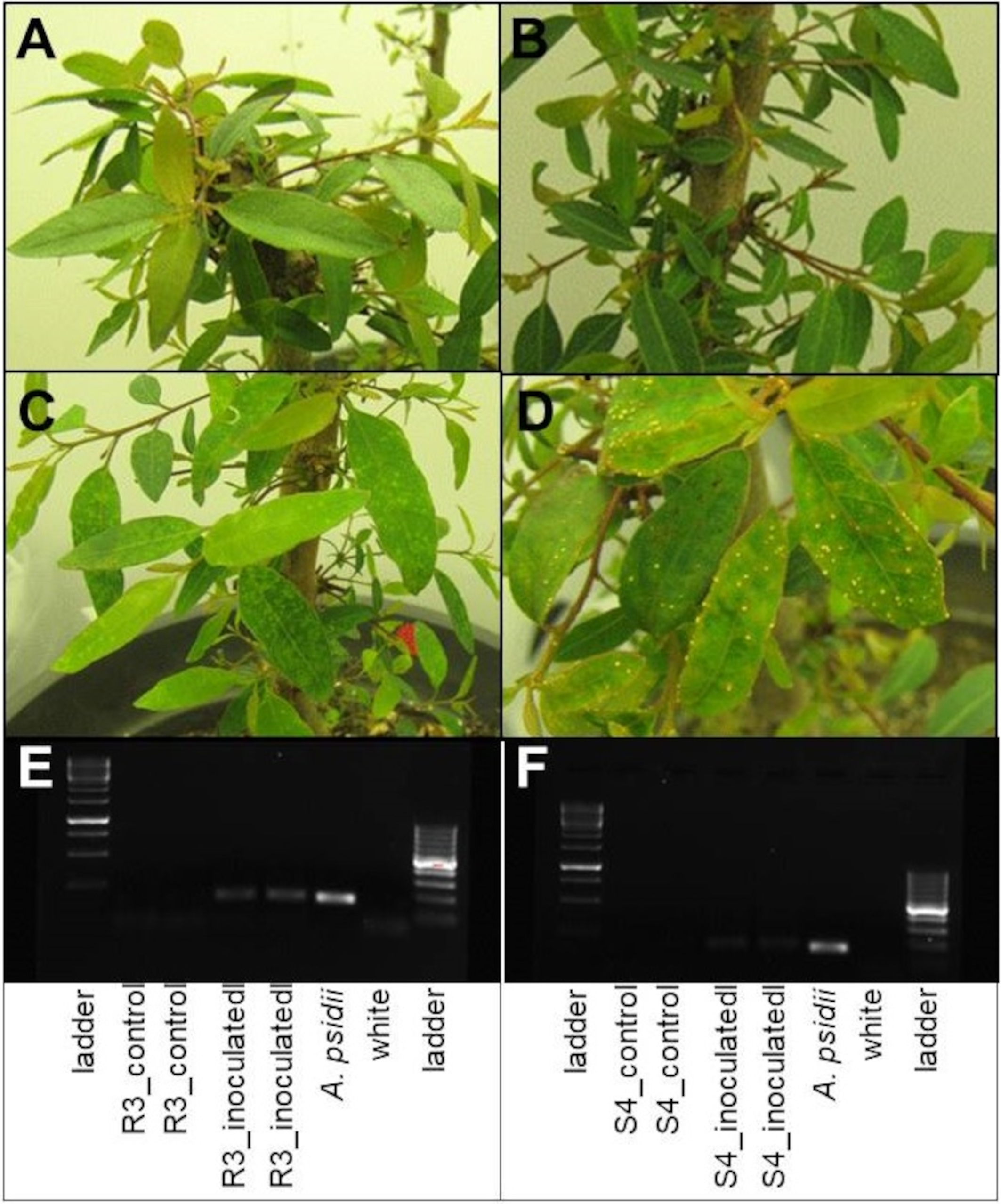

Supplement: Supplementary Figure 1 — Images of inoculation and control plants at 11 days after inoculation. (A) Control R3 and (B) S4 plants lack disease symptoms. (C) Inoculated R3 plants do not form pustules, but display symptoms of hypersensitive reactions. (D) Inoculated S4 plants have pustules covering the leaf surface. An agarose gel containing amplified DNA fragments produced using A. psidii-specific primer that confirm the inoculation of (E) R3 and (F) S4 genotypes. [file Image_1.JPEG]

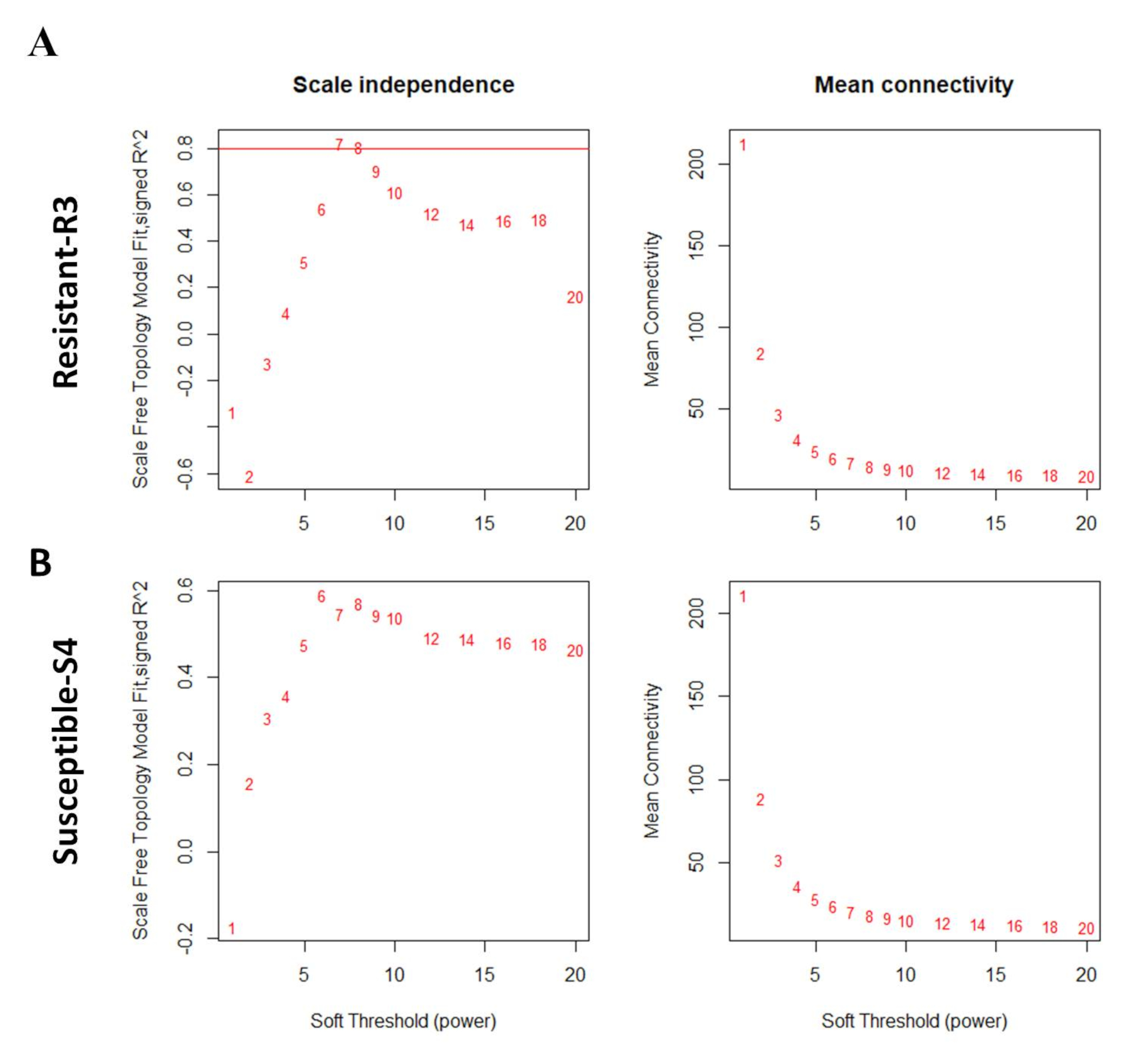

Supplement: Supplementary Figure 2 — Choosing soft threshold based on power value (numbers in red) for constructing adjacency matrix network. (A) Power 7 of resistant-R3 samples showed scale free topology value > 0.8 and mean connectivity < 50 and (B) power 6 of susceptible-S4 samples showed scale free topology value close to 0.6 and mean connectivity < 50. [file Image_2.JPEG]

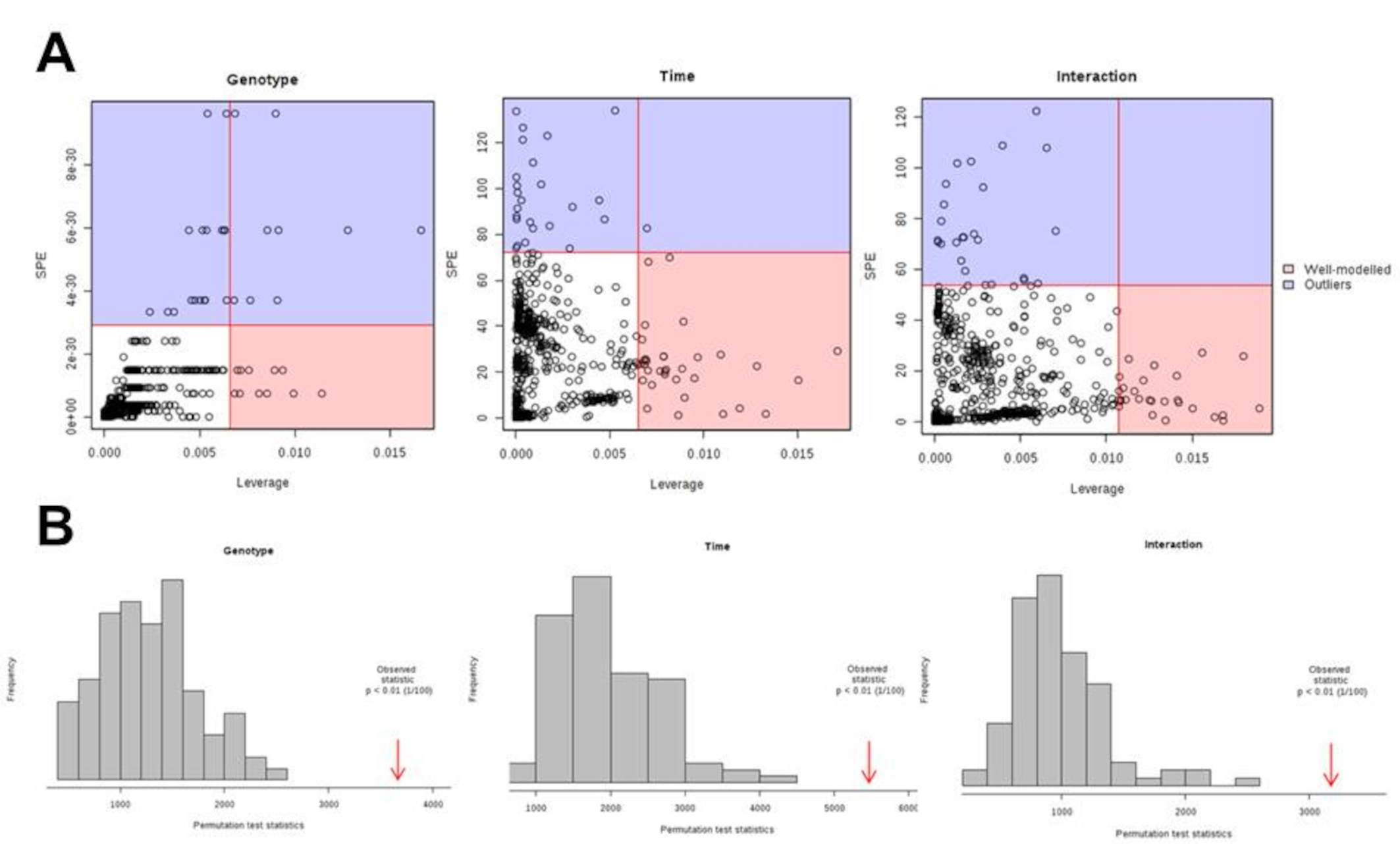

Supplement: Supplementary Figure 3 — Two-factor ASCA (ANOVA simultaneous component analysis) analysis for genotype, time-point and genotype vs. time-point interaction. (A) Distribution of metabolite features in which square predict error (SPE) < 0.05 and leverage > 0.9 for genotype for effects of time-point or genotype vs. time-point interactions. Outlier features are present in the upper quadrants (blue sections) and prominently regulated features are shown in the pink quadrant at the bottom right. (B) ASCA model validation. Distribution of sums of squares (SSQ) using 100x permutation of groups (genotype and time-points) is shown. Gray columns indicate SSQ distribution of data permutation and red arrows highlight the SSQ of original dataset format, which confirms the model. [file Image_3.JPEG]

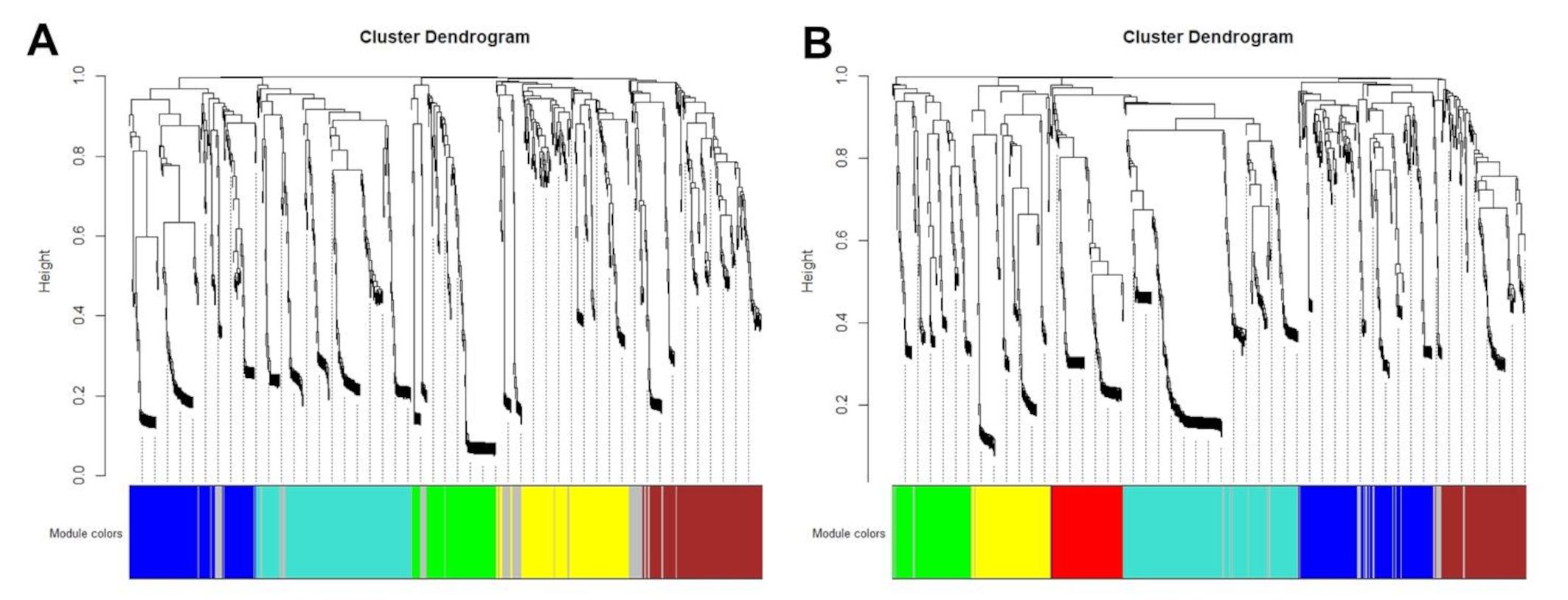

Supplement: Supplementary Figure 4 — WGCNA (weighted gene co-expression network analysis) cluster dendrogram of protein modules represented by color bars at the bottom. (A) Resistant-R3 genotype had 5 modules from 871 proteins. (B) Susceptible-S4 genotype has 6 modules from 852 proteins. [file Image_4.JPEG]
