# Supplementary material for: Network Analysis Combining Proteomics and Metabolomics Reveals New Insights Into Early Responses of Eucalyptus grandis During Rust Infection
Source: Front Plant Sci. 2021 Jan 7;11:604849. doi: 10.3389/fpls.2020.604849 (PMC7817549; doi:10.3389/fpls.2020.604849)
Supplement: Supplementary file 17 [file Data_Sheet_1.pdf]

MS/MS analysis Negative Mode

Database:
http://www.hmdb.ca/spectra/ms/search

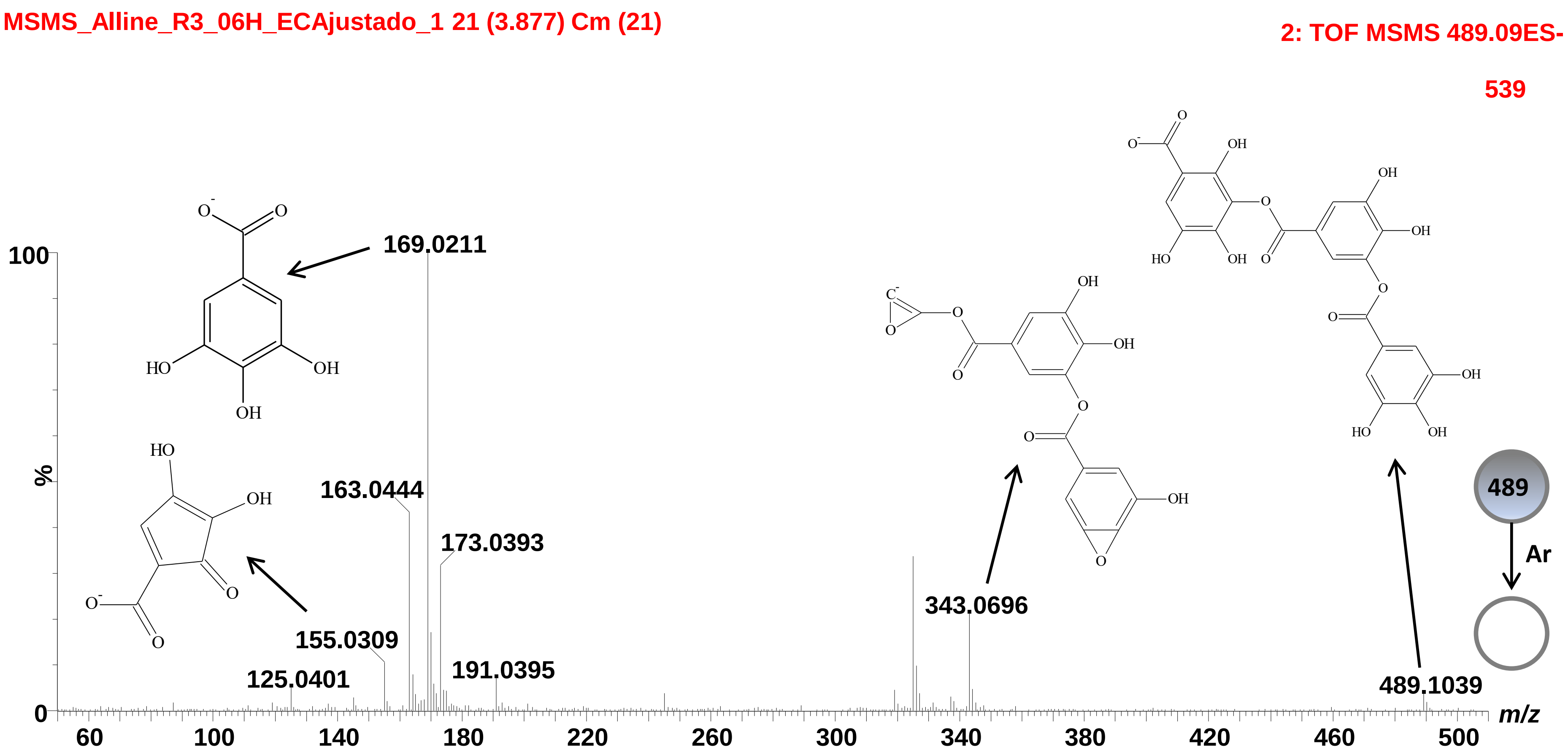

HMDB0128306
Class: Depsides and depsidones
Super class: Phenylpropanoids and polyketides

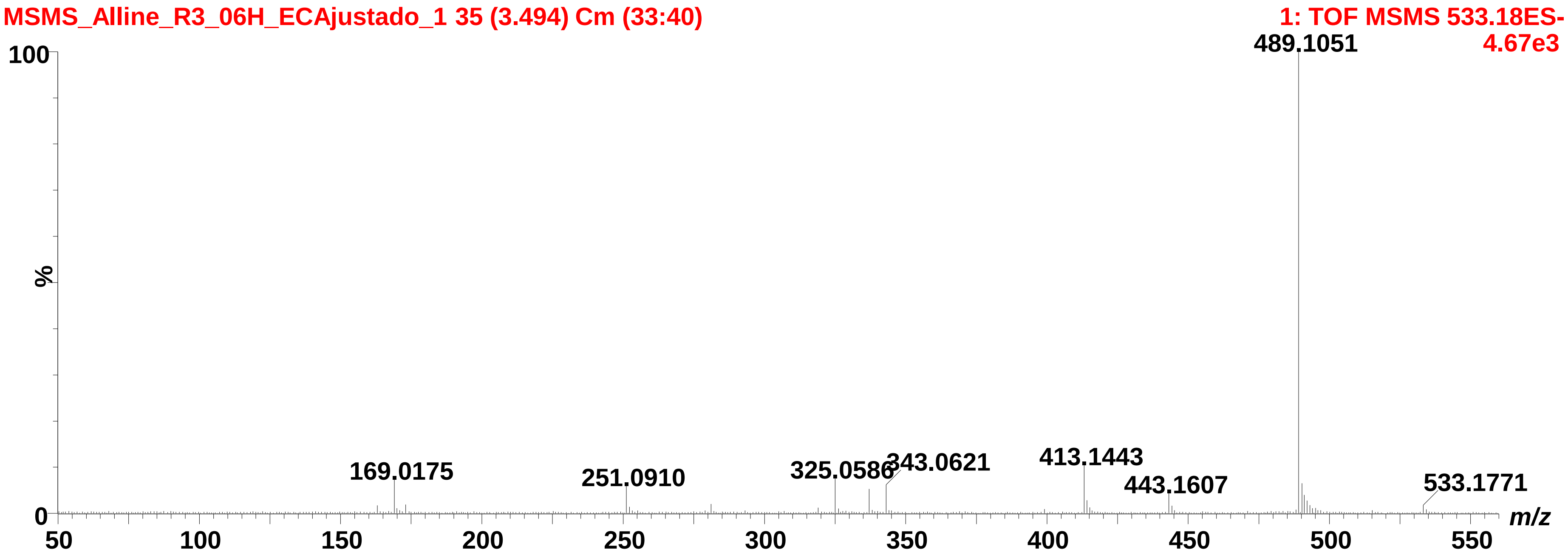
MSMS_Alline_R3_06H_ECAjustado_1 35 (3.494) Cm (33:40)
1: TOF MSMS 533.18ES-
4.67e3
489.1051
169.0175
251.0910
325.0586
343.0621
413.1443
443.1607
533.1771
100
%
0
50
100
150
200
250
300
350
400
450
500
550
m/z

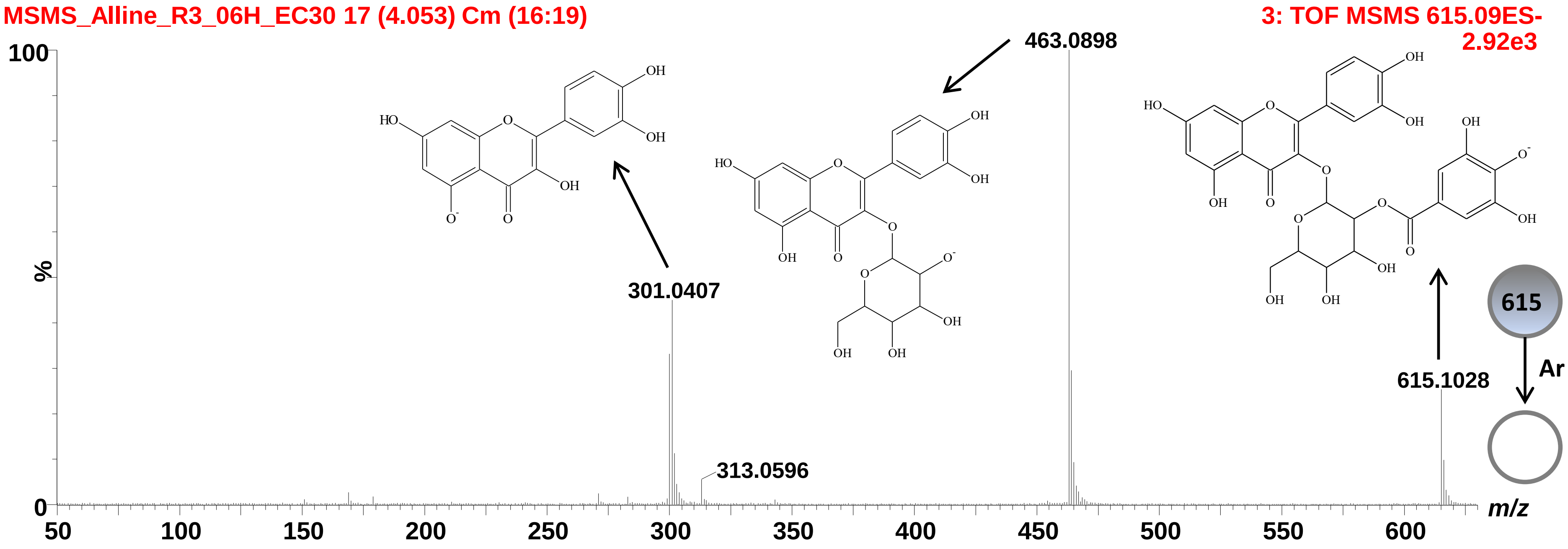

HMDB0037367

Class: Coumarans

Super class: Organoheterocyclic compounds

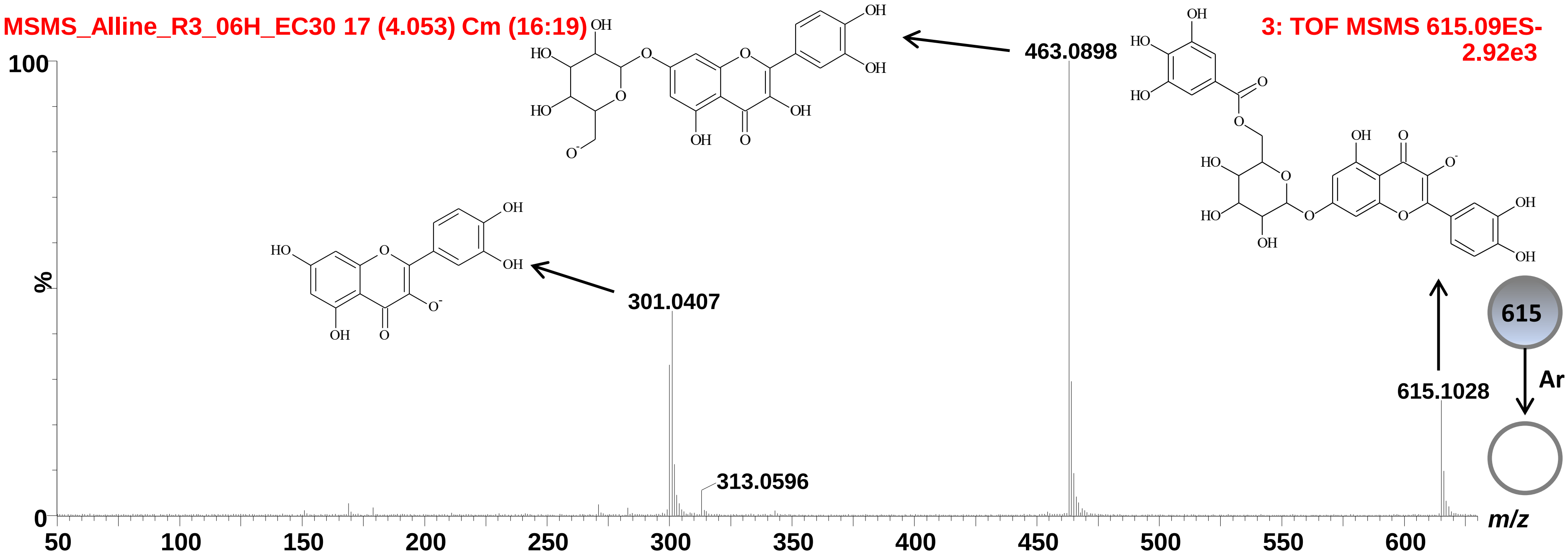

HMDB0033593

Class: Carboxylic acids and derivatives

Super class: Organic acids and derivatives

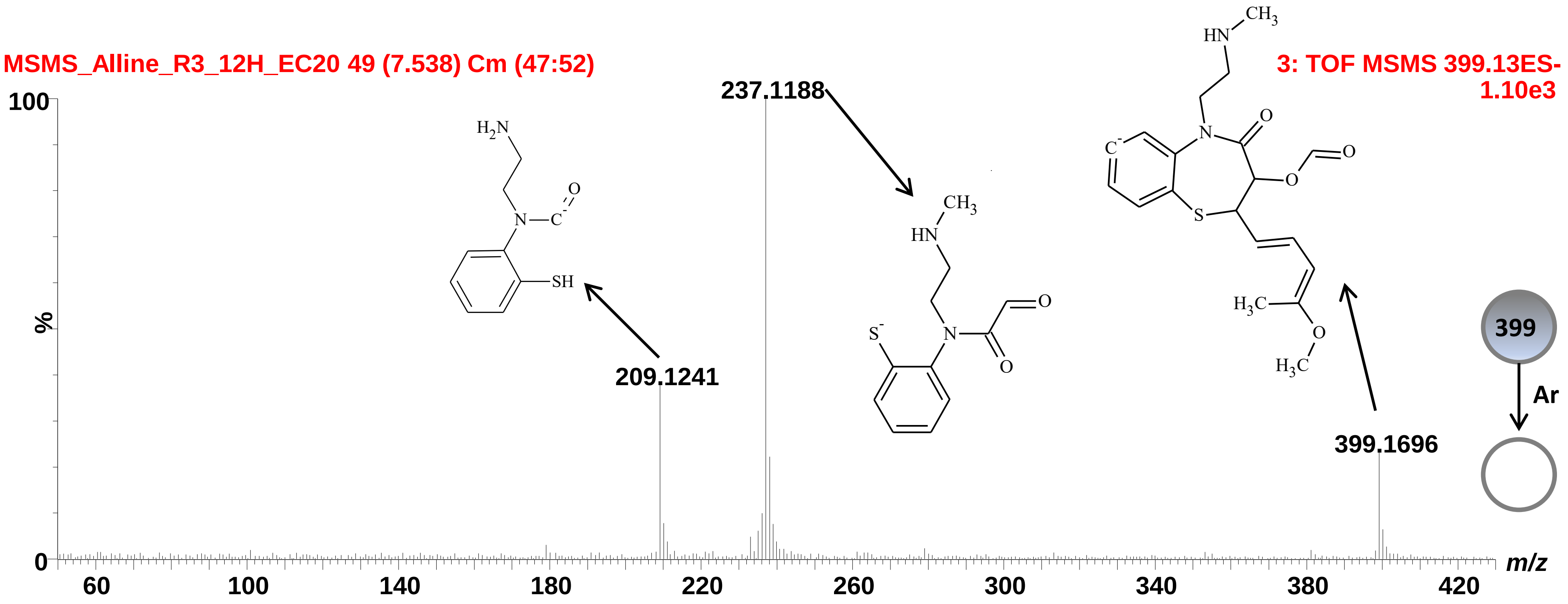

HMDB0061023

Class: Benzothiazepines

Super class: Organoheterocyclic compounds

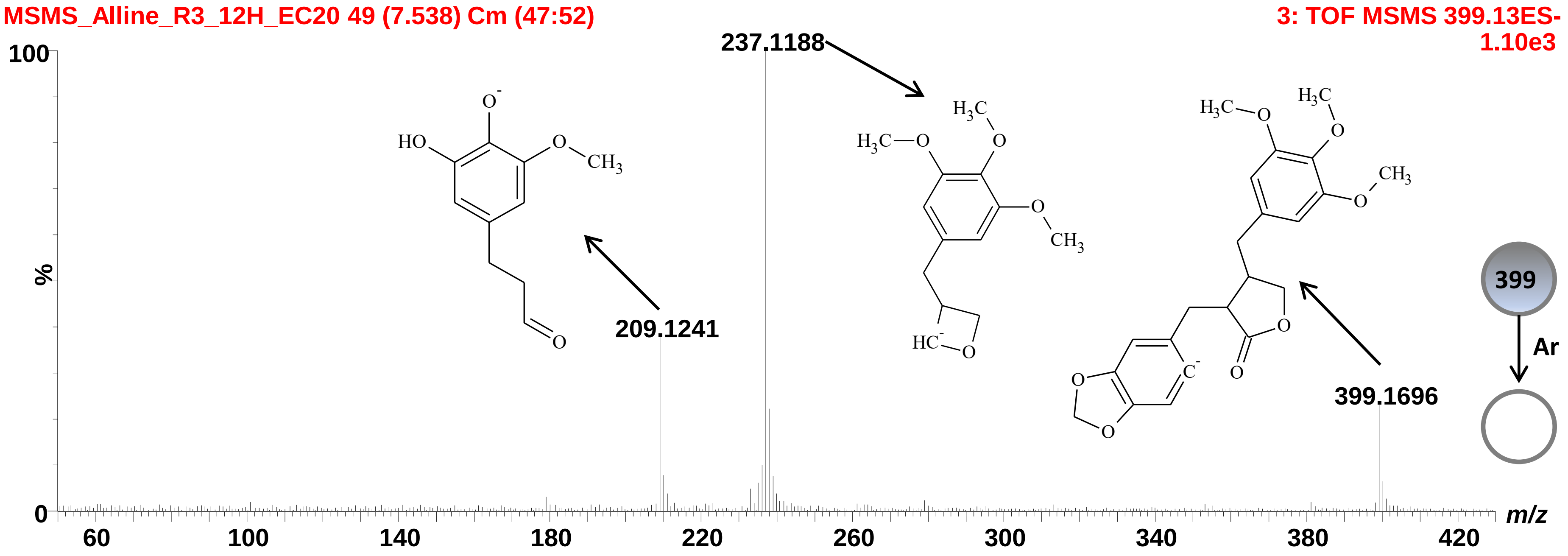

HMDB0033258

Class: Furanoid lignans

Super class: Lignans, neolignans and related compounds

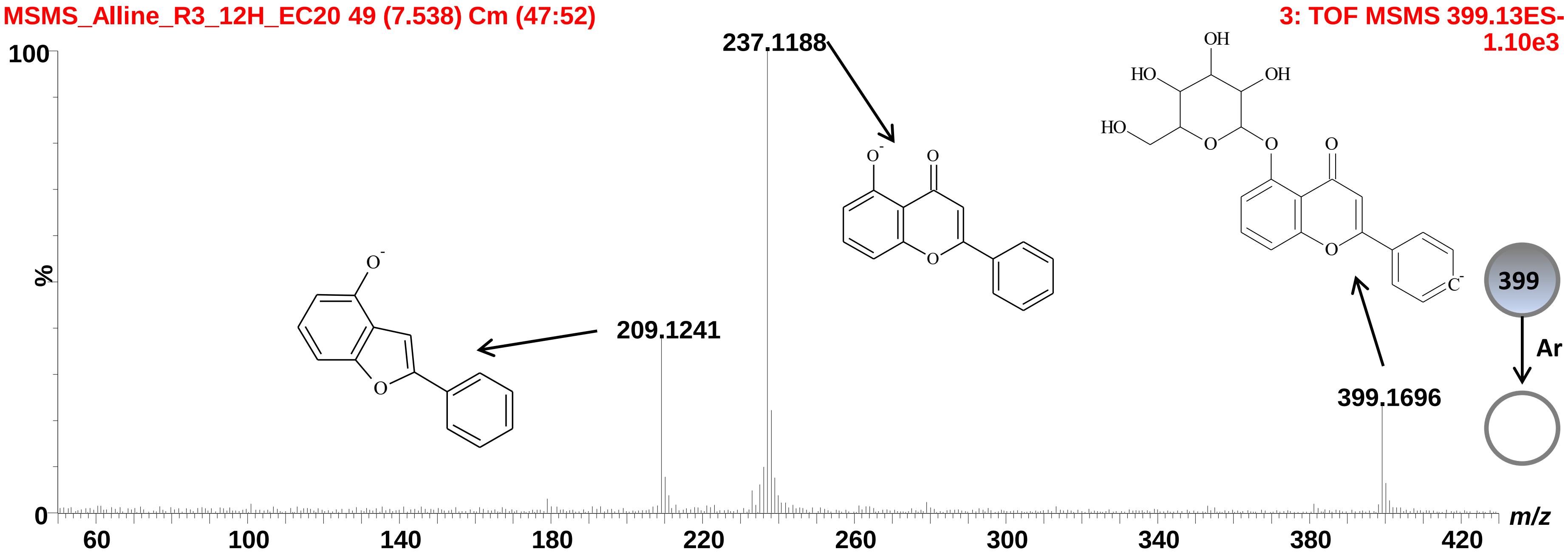

HMDB0040556

Class: Flavonoids

Super class: Phenylpropanoids and polyketides

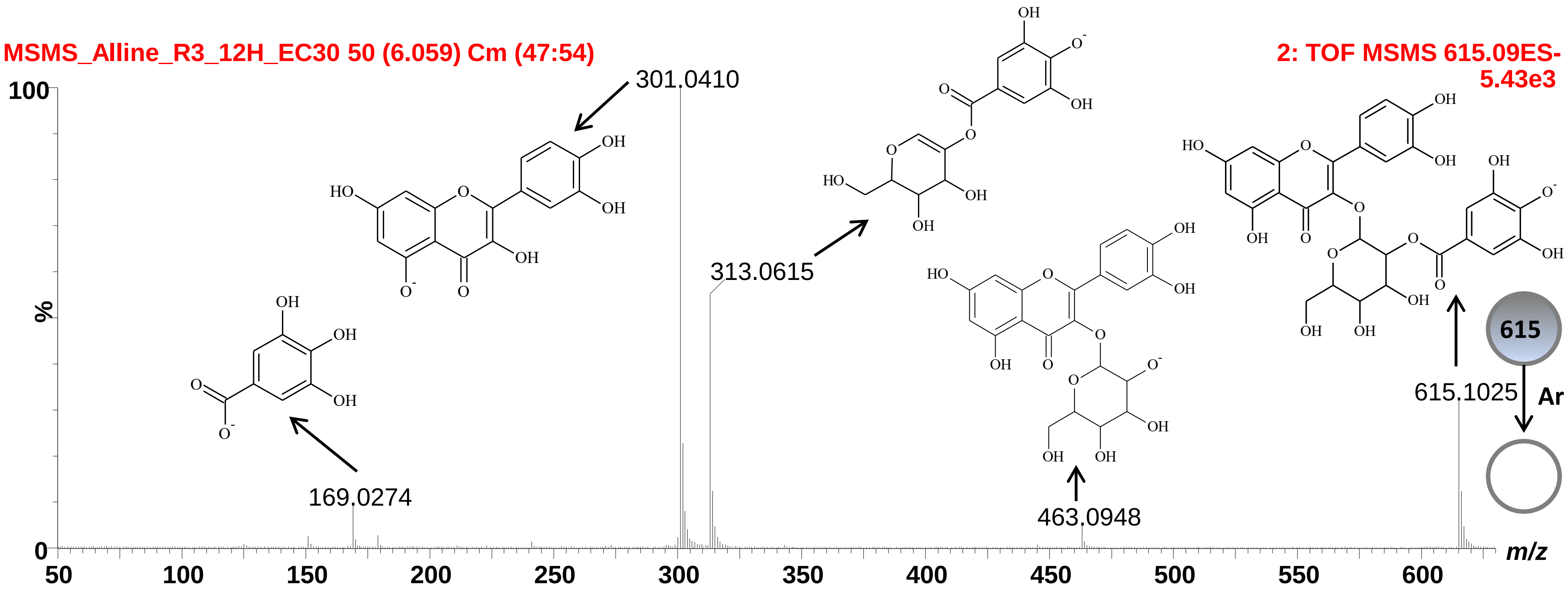

HMDB0037367

Class: Coumarans

Super class: Organoheterocyclic compounds

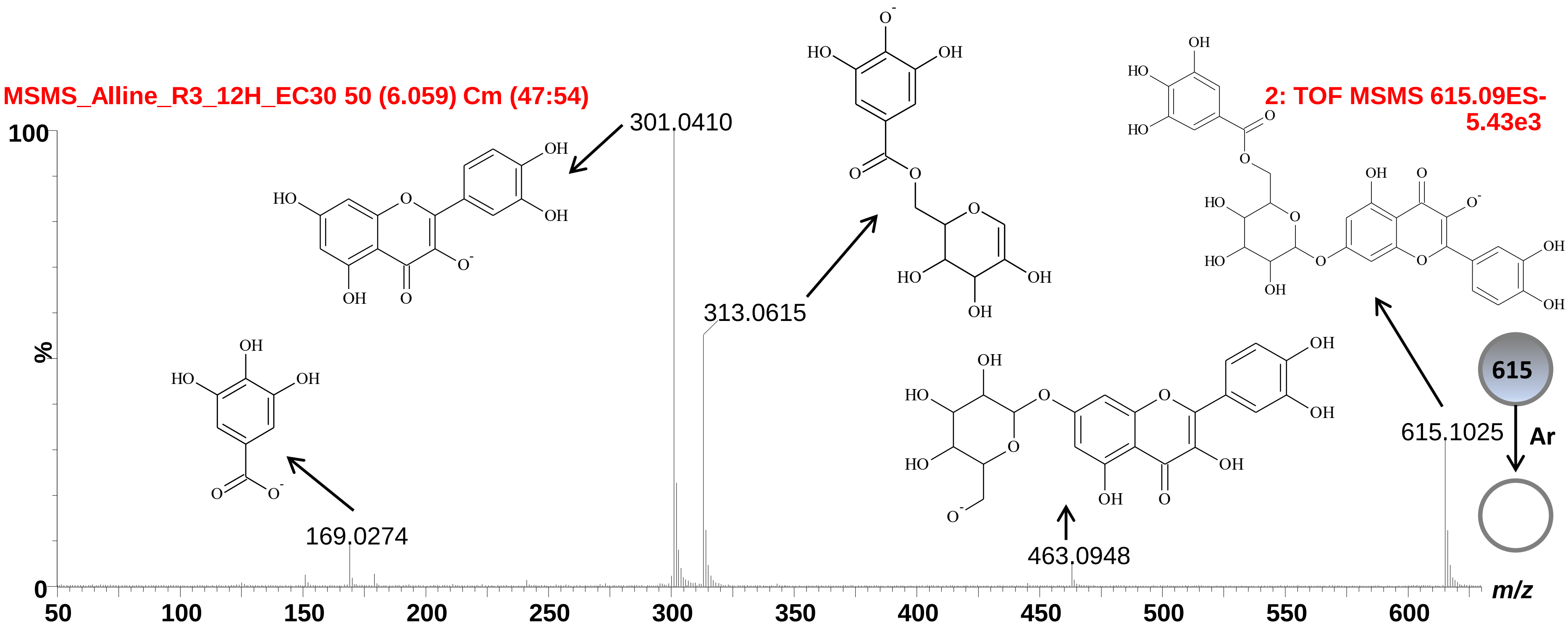

HMDB0033593

Class: Flavonoids

Super class: Phenylpropanoids and polyketides

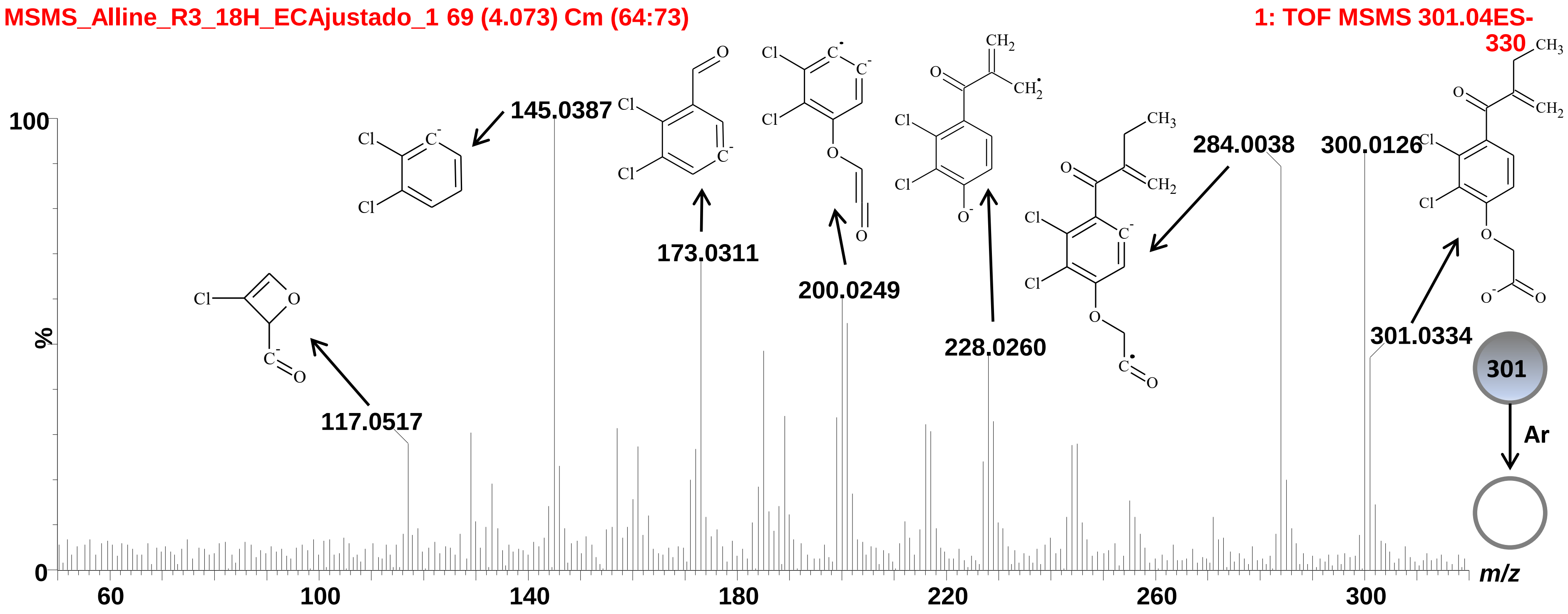

HMDB0015039

Class: Benzene and substituted derivatives

Super class: Benzenoids

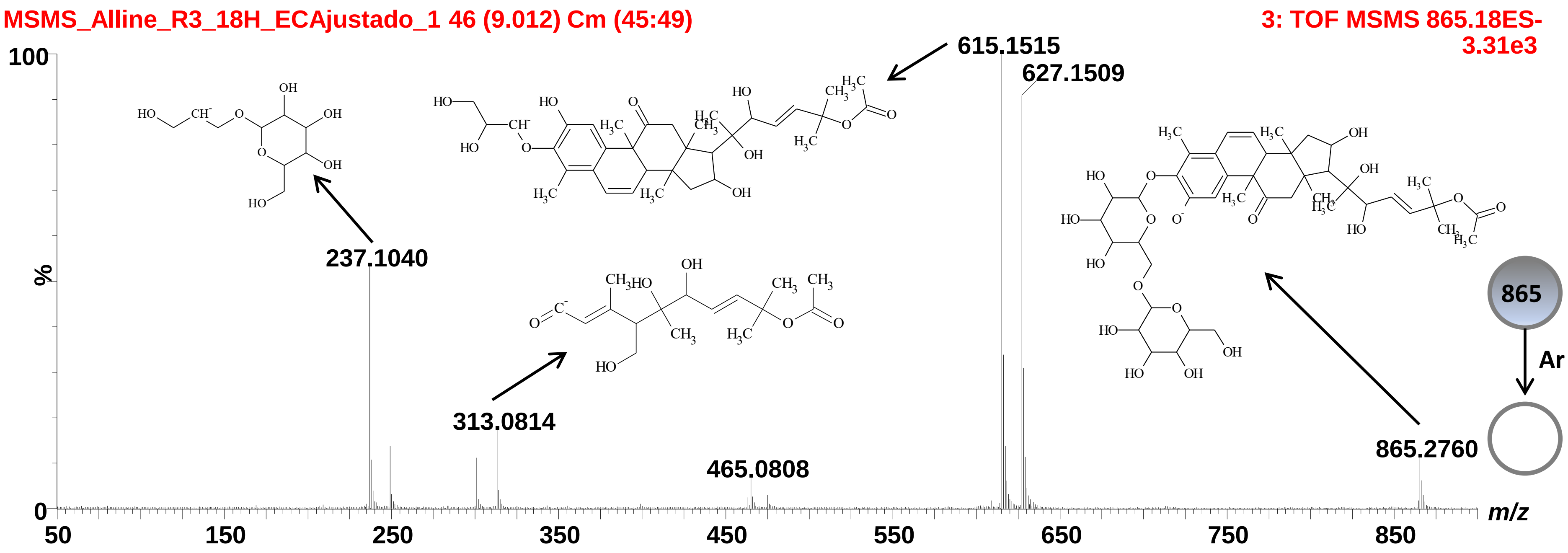

HMDB0036339

Class: Benzopyrans

Super class: Organoheterocyclic compounds

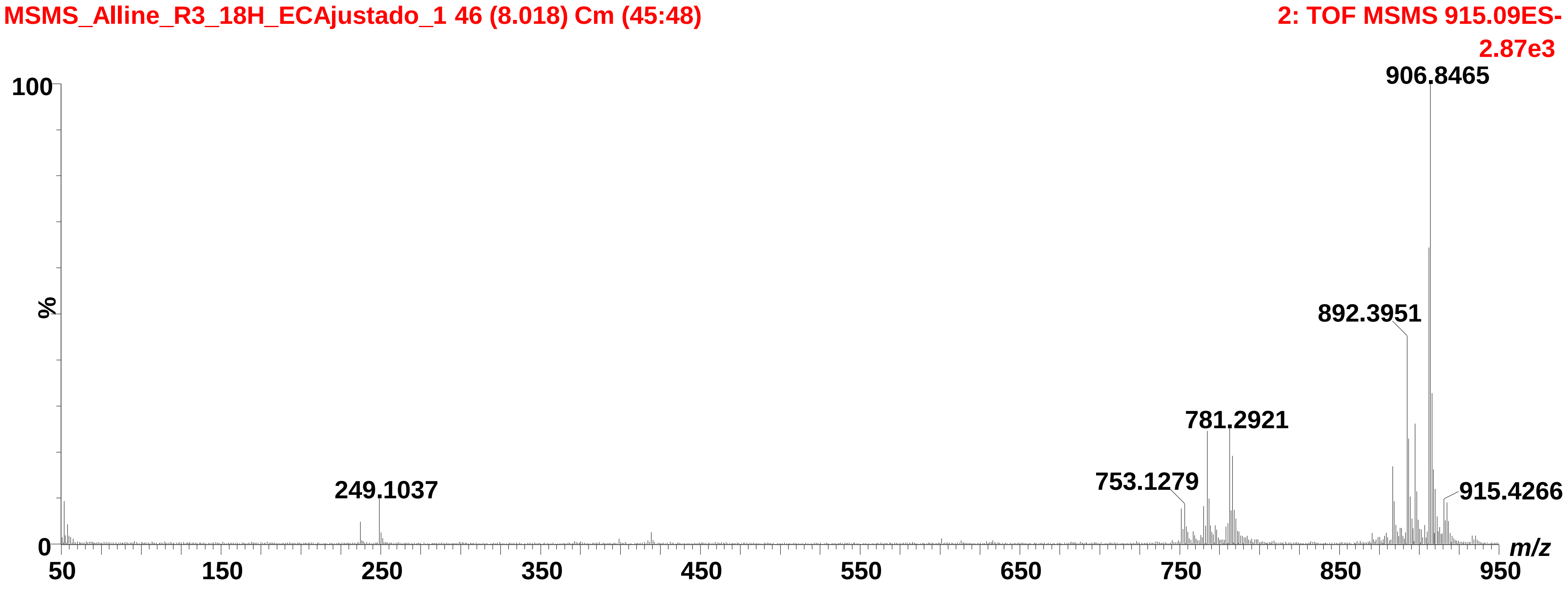

MSMS_Alline_R3_18H_ECAjustado_1 46 (8.018) Cm (45:48)
2: TOF MSMS 915.09ES-
2.87e3
100
%
0
906.8465
892.3951
781.2921
753.1279
915.4266
249.1037
50
150
250
350
450
550
650
750
850
950
m/z

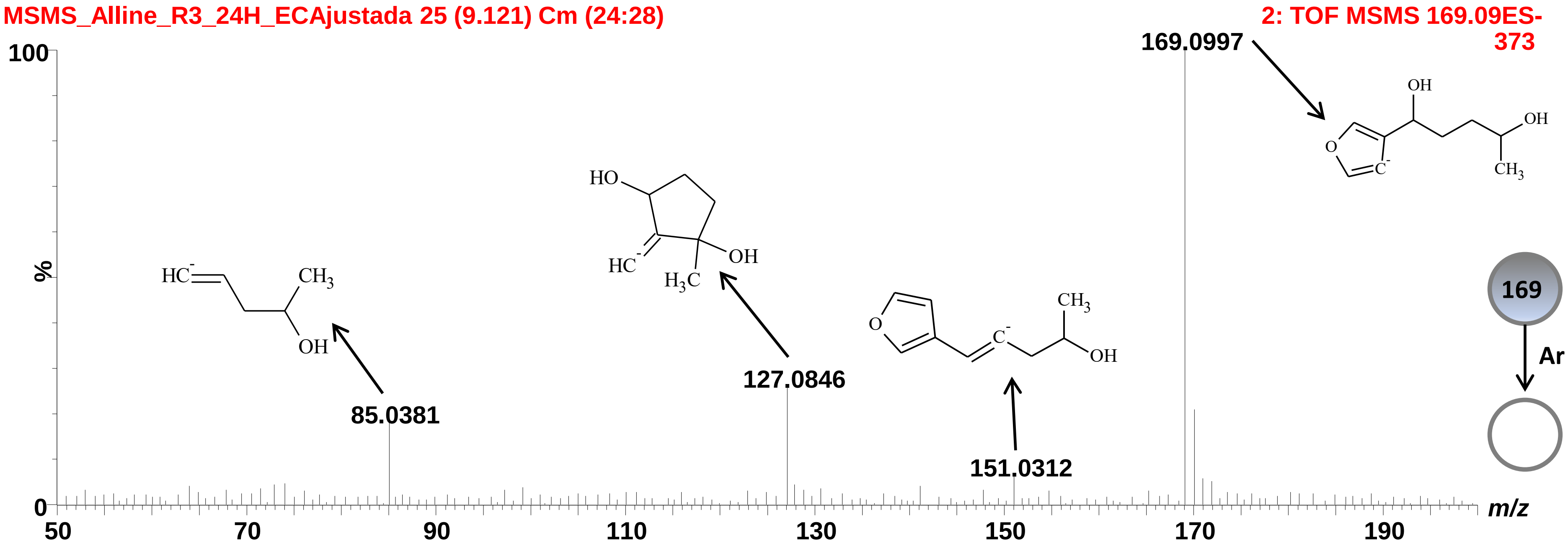

HMDB0030471

Class: Heteroaromatic compounds

Super class: Organoheterocyclic compounds

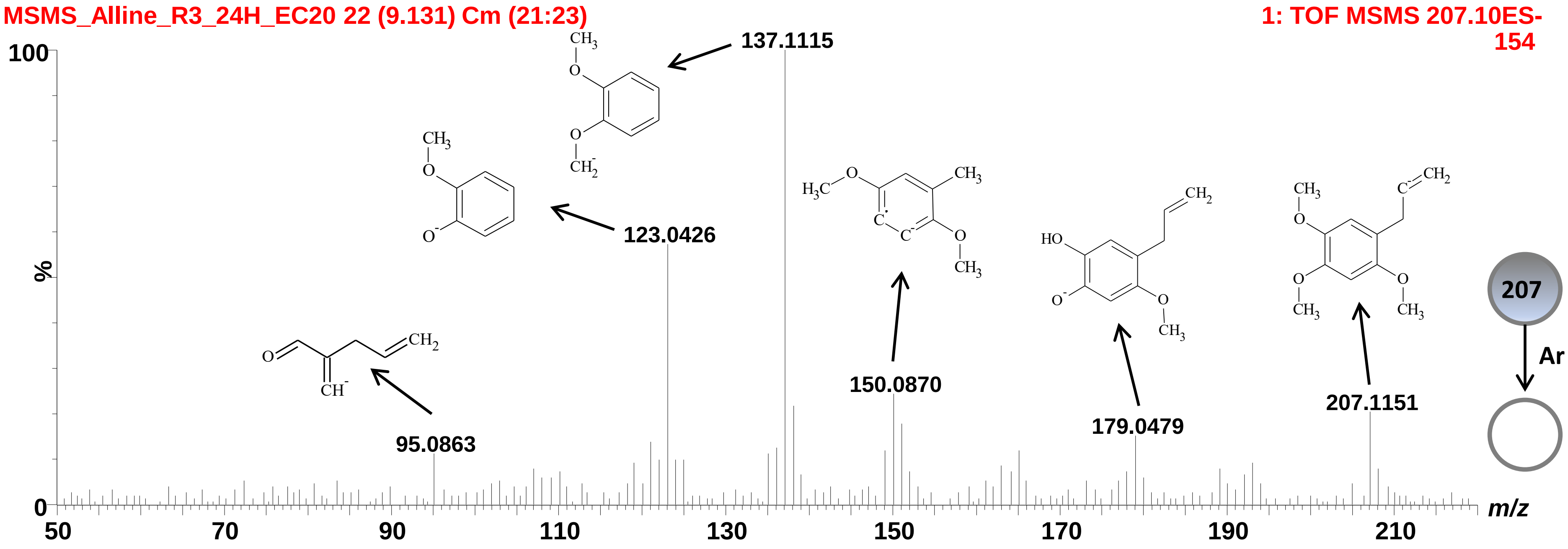

HMDB0029872

Class: Phenol ethers

Super class: Benzenoids

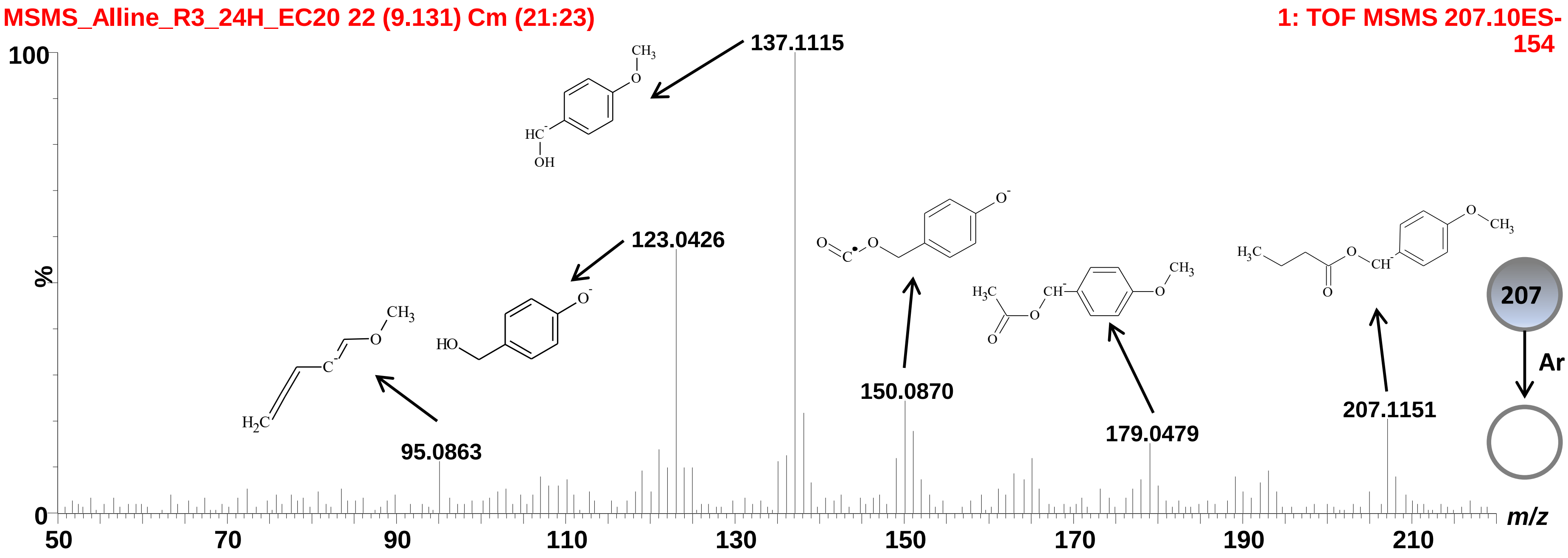

HMDB0034991

Class: Benzene and substituted derivatives

Super class: Benzenoids

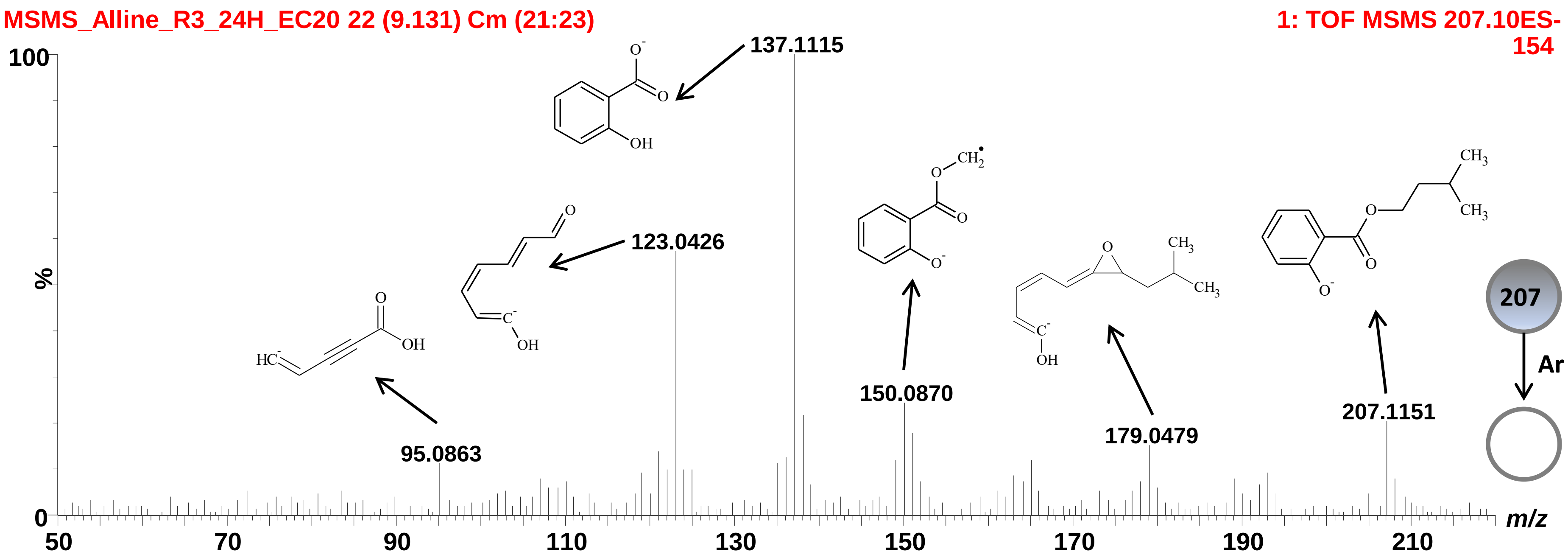

HMDB0040225

Class: Benzene and substituted derivatives

Super class: Benzenoids

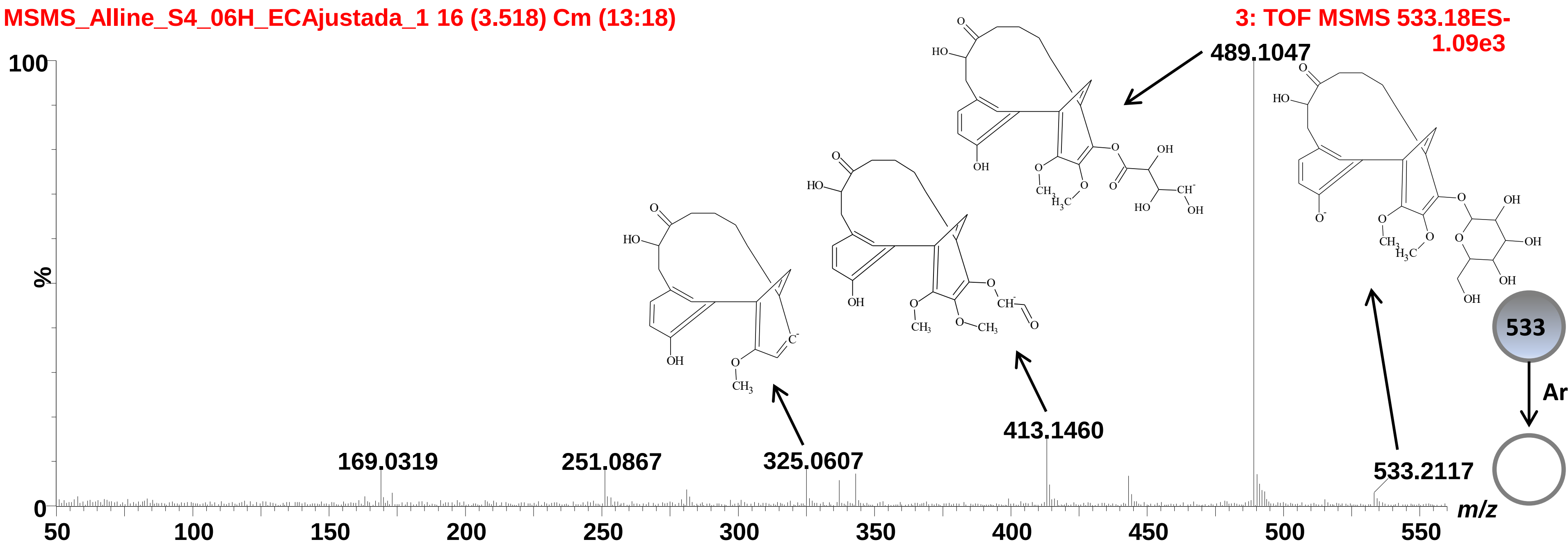

HMDB0031584

Class: Diarylheptanoids

Super class: Phenylpropanoids and polyketides

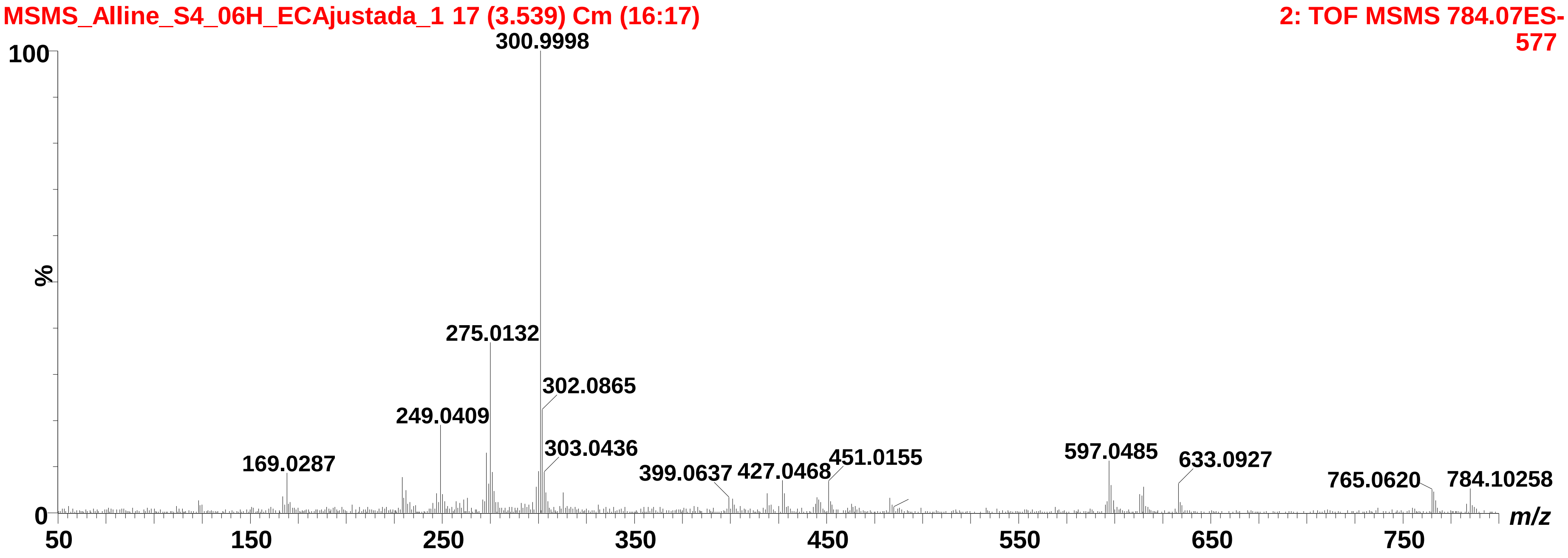
MSMS_Alline_S4_06H_ECAjustada_1 17 (3.539) Cm (16:17)
2: TOF MSMS 784.07ES-
577
100
%
0
300.9998
275.0132
302.0865
249.0409
303.0436
169.0287
399.0637
427.0468
451.0155
597.0485
633.0927
765.0620
784.10258
50
150
250
350
450
550
650
750
m/z

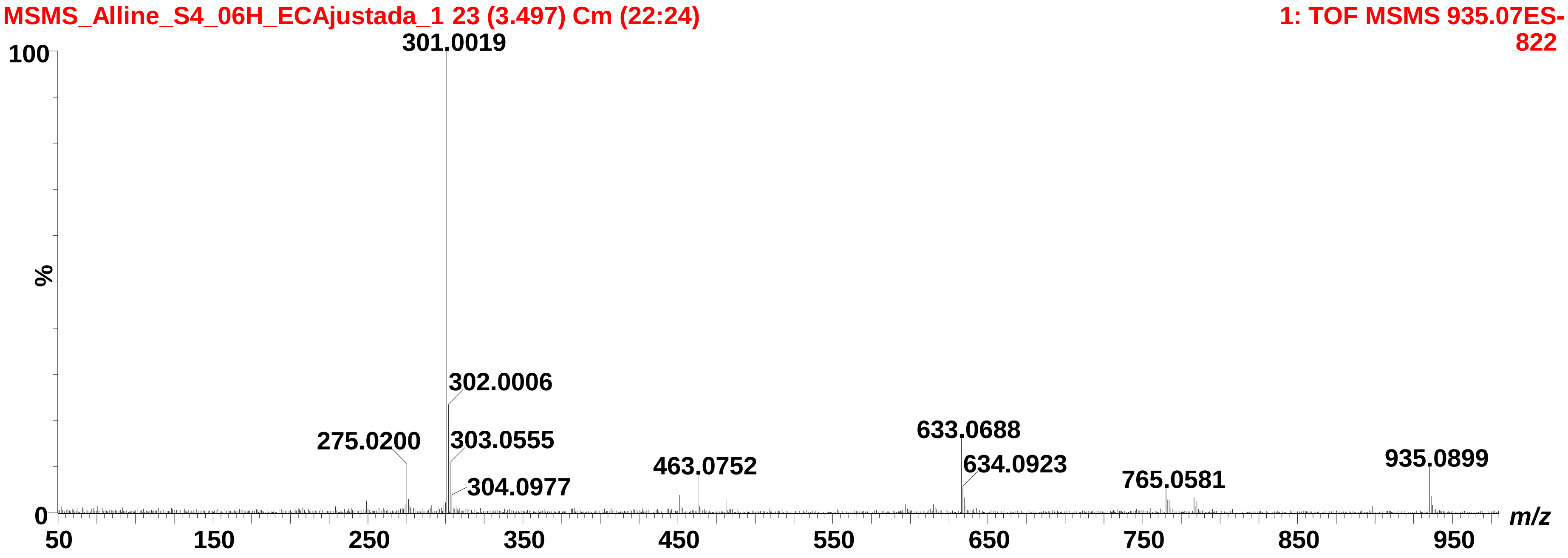
MSMS_Alline_S4_06H_ECAjustada_1 23 (3.497) Cm (22:24)
1: TOF MSMS 935.07ES-
822
100
%
0
301.0019
302.0006
275.0200
303.0555
304.0977
463.0752
633.0688
634.0923
765.0581
935.0899
50
150
250
350
450
550
650
750
850
950
m/z

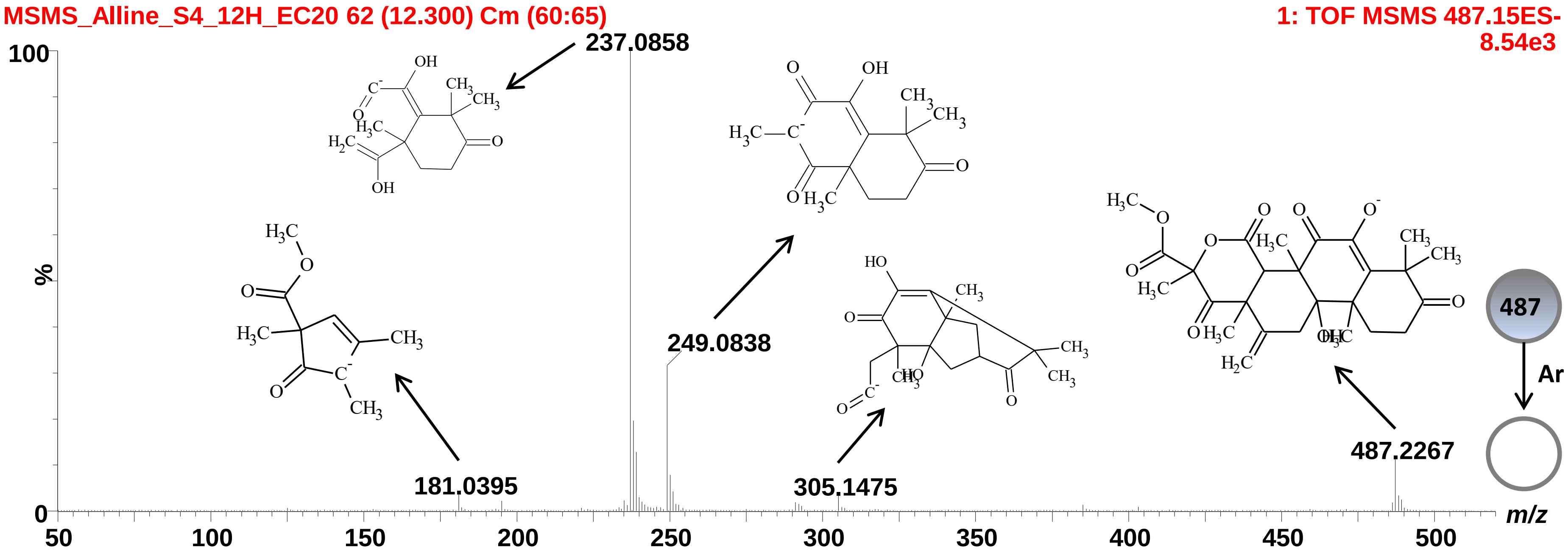

HMDB0033894

Class: Flavonoids

Super class: Phenylpropanoids and polyketides

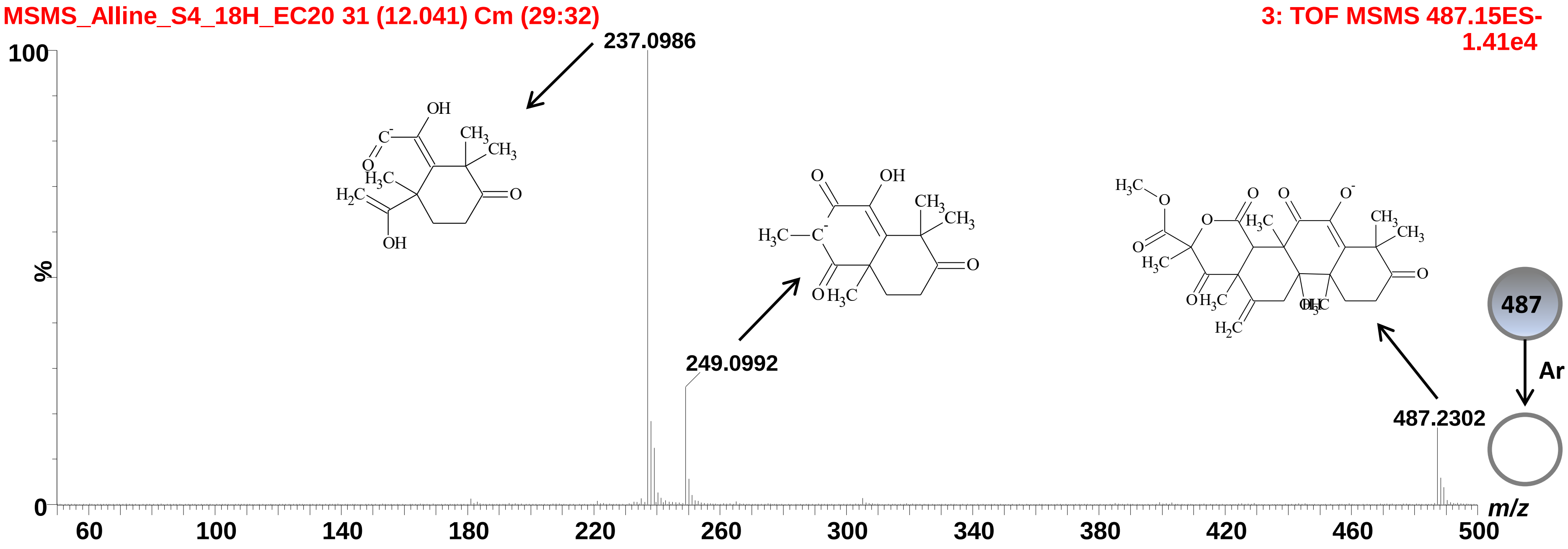

HMDB0033894

Class: Flavonoids

Super class: Phenylpropanoids and polyketides

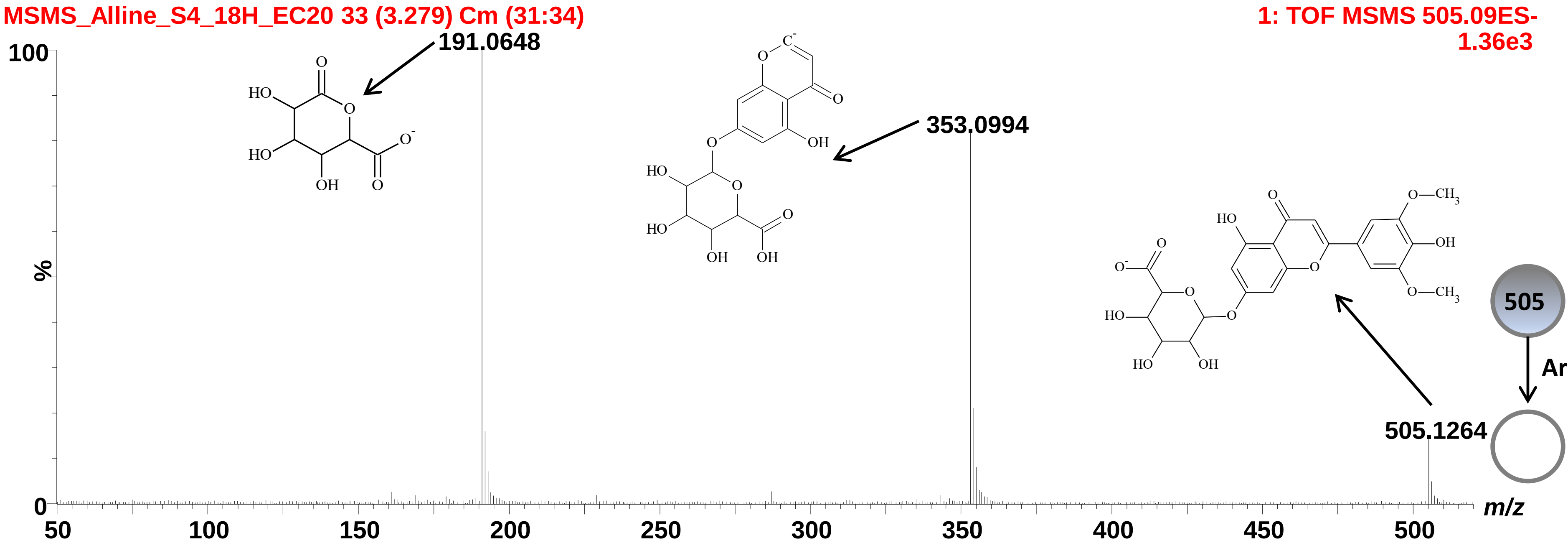

MSMS_Alline_S4_18H_EC20 33 (3.279) Cm (31:34)
1: TOF MSMS 505.09ES-
1.36e3
191.0648
353.0994
505.1264
505
Ar
100
%
0
50
100
150
200
250
300
350
400
450
500
m/z

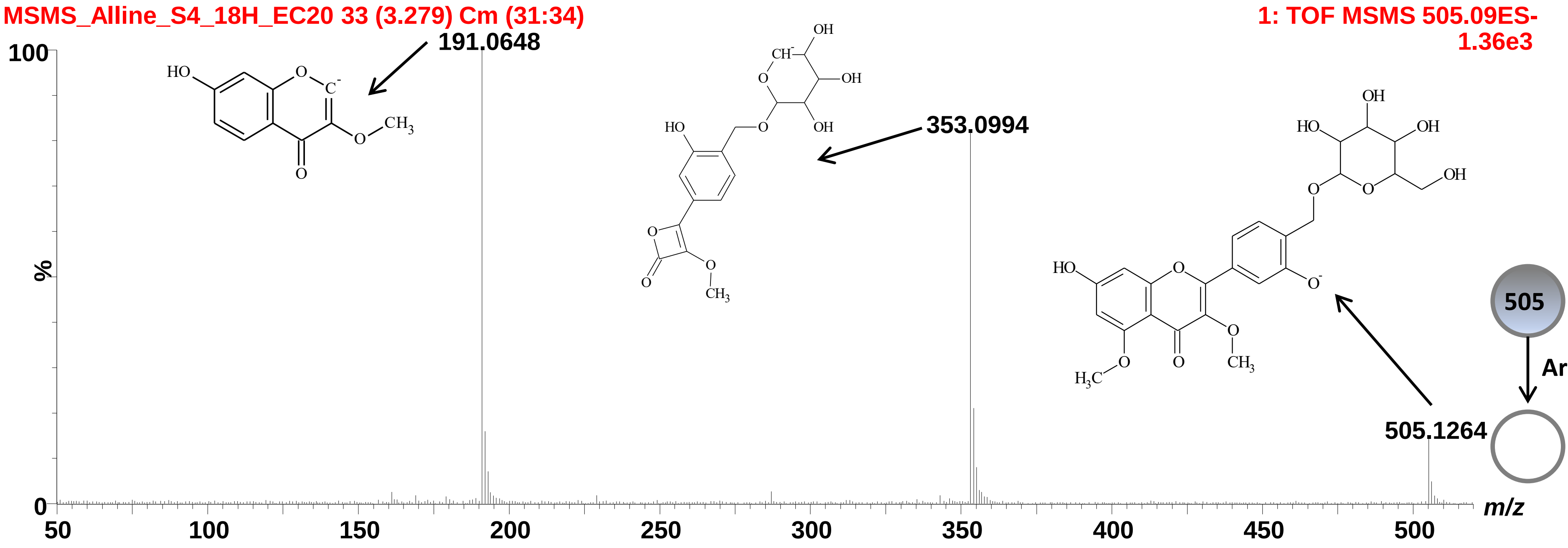

HMDB0037352

Class: Flavonoids

Super class: Phenylpropanoids and polyketides
